# In vivo evidence of ascorbate involvement in the generation of epigenetic DNA modifications in leukocytes from patients with colorectal carcinoma, benign adenoma and inflammatory bowel disease

**DOI:** 10.1186/s12967-018-1581-9

**Published:** 2018-07-20

**Authors:** Marta Starczak, Ewelina Zarakowska, Martyna Modrzejewska, Tomasz Dziaman, Anna Szpila, Kinga Linowiecka, Jolanta Guz, Justyna Szpotan, Maciej Gawronski, Anna Labejszo, Ariel Liebert, Zbigniew Banaszkiewicz, Maria Klopocka, Marek Foksinski, Daniel Gackowski, Ryszard Olinski

**Affiliations:** 1 Department of Clinical Biochemistry, Faculty of Pharmacy, Collegium Medicum in Bydgoszcz, Nicolaus Copernicus University in Toruń, Karlowicza 24, 85-092 Bydgoszcz, Poland; 2 Department of Vascular Surgery and Angiology, Faculty of Medicine, Collegium Medicum in Bydgoszcz, Nicolaus Copernicus University in Toruń, M. Curie Sklodowskiej 9, 85-094 Bydgoszcz, Poland; 3 Department of Vascular Diseases and Internal Medicine, Faculty of Health Sciences, Collegium Medicum in Bydgoszcz, Nicolaus Copernicus University in Toruń, Ujejskiego 75, 85-168 Bydgoszcz, Poland; 4 Department of General, Gastrointestinal, Colorectal and Oncological Surgery, Jan Biziel University No. 2 in Bydgoszcz, Ujejskiego 75, 85-168 Bydgoszcz, Poland

**Keywords:** Epigenetic DNA modifications, DNA demethylation, Colorectal cancer, IBD, Colon polyp, Ascorbate

## Abstract

**Background:**

A characteristic feature of malignant cells, such as colorectal cancer cells, is a profound decrease in the level of 5-hydroxymethylcytosine, a product of 5-methylcytosine oxidation by TET enzymes. Recent studies showed that ascorbate may upregulate the activity of TET enzymes in cultured cells and enhance formation of their products in genomic DNA.

**Methods:**

The study included four groups of subjects: healthy controls (n = 79), patients with inflammatory bowel disease (IBD, n = 51), adenomatous polyps (n = 67) and colorectal cancer (n = 136). The list of analyzed parameters included (i) leukocyte levels of epigenetic DNA modifications and 8-oxo-7,8-dihydro-2′-deoxyguanosine, a marker of oxidatively modified DNA, determined by means of isotope-dilution automated online two-dimensional ultra-performance liquid chromatography with tandem mass spectrometry, (ii) expression of TET mRNA measured with RT-qPCR, and (iii) chromatographically-determined plasma concentrations of retinol, alpha-tocopherol and ascorbate.

**Results:**

Patients from all groups presented with significantly lower levels of 5-methylcytosine and 5-hydroxymethylcytosine in DNA than the controls. A similar tendency was also observed for 5-hydroxymethyluracil level. Patients with IBD showed the highest levels of 5-formylcytosine and 8-oxo-7,8-dihydro-2′-deoxyguanosine of all study subjects, and individuals with colorectal cancer presented with the lowest concentrations of ascorbate and retinol. A positive correlation was observed between plasma concentration of ascorbate and levels of two epigenetic modifications, 5-hydroxymethylcytosine and 5-hydroxymethyluracil in leukocyte DNA. Moreover, a significant difference was found in the levels of these modifications in patients whose plasma concentrations of ascorbate were below the lower and above the upper quartile for the control group.

**Conclusions:**

These findings suggest that deficiency of ascorbate in the blood may be a marker of its shortage in other tissues, which in turn may correspond to deterioration of DNA methylation-demethylation. These observations may provide a rationale for further research on blood biomarkers of colorectal cancer development.

**Electronic supplementary material:**

The online version of this article (10.1186/s12967-018-1581-9) contains supplementary material, which is available to authorized users.

## Background

Mutations and aberrant methylation patterns are generally accepted as early events and important determinants of colon carcinogenesis. However, observed differences in the incidence of colon cancer seem to result primarily from the influence of environmental factors, among them oxidative stress which may be also linked to epigenetic changes [1–4].

Methylation of cytosine, a key epigenetic modification, usually involving CpG dinucleotides, is closely linked to gene repression, a process that exerts a profound effect on cellular identity and organismal fate [5]. Equally important is active DNA demethylation, a recently discovered process which results in activation of previously silenced genes. Molecular background of active DNA demethylation is still not completely understood (reviewed in [6]). The most plausible mechanism involves ten-eleven translocation (TET) proteins that catalyze oxidization of 5-methylcytosine (5-mCyt) to 5-hydroxymethylcytosine (5-hmCyt), and then to 5-formylcytosine (5-fCyt) which is eventually converted to 5-carboxycytosine (5-caCyt) [6, 7]. Some evidence from experimental studies suggests that TETs may be also involved in synthesis of 5-hydroxymethyluracil (5-hmUra), a compound with epigenetic function [8].

A plethora of recent studies demonstrated unequivocally that 5-hmCyt is profoundly reduced in many types of human malignancies, including colorectal cancer [9–11]. However, it is still unclear whether this phenomenon is limited solely to tumor tissue, or may occur also in surrogate materials from cancer patients, for example, leukocytes.

Moreover, it cannot be excluded that active DNA demethylation taking place under altered conditions or in a different environment, for example in presence of chronic inflammation (that may induce oxidative stress) or in malnutrition (that may influence ascorbate level), may modulate TET activity and thus, affect the level of epigenetic modifications.

Our previous research demonstrated that colorectal cancer patients present with significantly (ca. 30%) reduced levels of ascorbate [12, 13]. Recent studies showed that ascorbate may enhance generation of 5-hmCyt in cultured cells [14–17]. Also retinol has been demonstrated recently to enhance the synthesis of 5-hmCyt and to modulate the level of TETs [18]. Consequently, it cannot be excluded that the level of epigenetic DNA modifications and the expression of TETs in leukocytes are associated with blood concentrations of ascorbate and retinol.

In this study, we used our recently developed rapid, highly-sensitive and highly-specific isotope-dilution automated online two-dimensional ultra-performance liquid chromatography with tandem mass spectrometry (2D-UPLC-MS/MS) [19, 20] to analyze global methylation and to determine the levels of TET-mediated oxidation products of 5-mCyt and thymine: 5-hmCyt, 5-fCyt, 5-caCyt and 5-hmUra. Moreover, we analyzed the level of the best characterized marker of oxidatively modified DNA, 8-oxo-7,8-dihydro-2′-deoxyguanosine (8-oxodG), as well as the expression of TETs mRNA, and plasma concentrations of antioxidant vitamins: ascorbate, retinol and α-tocopherol.

Obtaining altered tissues from patients with some pathological conditions may be challenging. However, some studies demonstrated that the analysis of non-affected tissues can provide equally informative results (reviewed in [21]). Leukocytes are often used as an easily accessible cells carrying information about environmentally-induced DNA modifications in other tissues [22, 23].

Despite the fact that metabolic changes closely linked to inflammation may influence 5-hmCyt and formation of its derivatives, none of the previous studies analyzed the effect of chronic inflammation on the generation of 5-hmCyt derivatives in leukocytes. Our study may also fill another knowledge gap, demonstrating how various conditions predisposing to colorectal cancer can shape TET-mediated DNA modifications and oxidatively modified DNA in an easily accessible tissue, leukocytes.

In this study, we examined leukocytes from colorectal cancer patients and individuals with two most common conditions predisposing to sporadic colorectal malignancies, colon polyps and inflammatory bowel disease (IBD).

## Methods

### Study group

The study included four groups of subjects: (1) healthy controls (n = 79, median age 55 years, 63% of women), (2) patients with IBD (n = 51, median age 35 years, 53% of women), (3) persons with adenomatous polyps, i.e. histologically confirmed adenoma tubulare (90%) or adenoma tubulovillosum (10%) (n = 67, median age 65 years, 46% of women), and (4) individuals with colorectal cancer, i.e. histologically confirmed stage A (8%), stage B (45%), stage C (29%) or stage D (9%) adenocarcinoma, or Hagitt scale I–IV carcinoma (9%) (n = 136, median age 65 years, 46% of women). None of the study subjects were related with one another, and all of them were Caucasians. All participants of the study were recruited in a hospital setting (Collegium Medicum, Nicolaus Copernicus University, Bydgoszcz, Poland) and subjected to colonoscopy. The study groups were matched for dietary habits, body weight and smoking status. The protocol of the study was approved by the Local Bioethics Committee, Collegium Medicum in Bydgoszcz, Nicolaus Copernicus University in Torun (Poland), and written informed consent was sought from all the participants.

### Isolation of DNA and determination of epigenetic modifications and 8-oxodG in DNA isolates

Leukocytes were isolated from heparinized blood samples with Histopaque 1119 (Sigma) solution, according to the manufacturer’s instruction, and stored at − 80 °C until the analysis. Isolation of leukocyte DNA and its hydrolysis were carried out as previously described [20], but the cell pellet was immediately dispersed in ice-cold lysing buffer B, without homogenization and washing steps. Procedures used for the determination of 5-methyl-2′-deoxycytidine (5-mdC), 5-(hydroxymethyl)-2′-deoxycytidine (5-hmdC), 5-formyl-2′-deoxycytidine (5-fdC), 5-carboxy-2′deoxycytidine (5-cadC), 5-(hydroxymethyl)-2′-deoxyuridine (5-hmdU) and 8-oxodG by 2D-UPLC-MS/MS have been described elsewhere [20]. Transition patterns, specific detector settings for all analyzed compounds are presented in Additional file 1: Table S1.

### Determination of ascorbate in blood plasma by UPLC-UV

#### Sample preparation

To stabilize ascorbate and to precipitate proteins, 200-µL aliquots of freshly prepared or partially thawed plasma were mixed with 200 µL of precooled 10% (w/v) meta-phosphoric acid (MPA, Sigma-Aldrich, Munich, Germany) containing uracil (50 µM, Sigma-Aldrich) as an internal standard. The samples were kept on ice for 40 min and then diluted with 200 µL of MilliQ-grade deionized water (Merck Millipore), vortexed and centrifuged at 25,155×*g* for 15 min at 4 °C. The supernatants (200 µL) were purified by ultrafiltration using AcroPrep Advance 96-Well Filter Plates 10 K (Pall), and injected into Waters Acquity ultra-performance liquid chromatographic (UPLC) system. The method was validated with the reference material from Chromsystems.

#### Chromatography

The UPLC system consisted of binary solvent manager, sample manager, column manager and photo-diode array detector, all from Waters. The samples were separated on Waters Acquity UPLC HSS T3 column (150 mm × 2.1 mm, 1.8 µm) with Van Guard HSS T3 1.8-µm pre-column at a flow rate 0.25 mL/min and 2-µL injection volume. Ammonium formate (10 mM, pH 3.1, Fluka) and acetonitrile (Sigma-Aldrich) were used as Solvent A and B, respectively. The following program was used for ascorbate elution: 0–0.1 min 99% A, 1% B, 0.1–2.2 min 97% A, 2.2–4.0 min—linear gradient to 90% A, 4.0–4.5 min—90% A, 4.5–6.0 min—99% A. Column thermostat was set at 10 °C. The effluent was monitored with a photo-diode array detector at 245 nm, and analyzed with Empower software.

### Determination of retinol and α-tocopherol in blood plasma by HPLC-FD

#### Sample preparation

To precipitate proteins, 200-µL aliquots of freshly thawed plasma were mixed with 200 µL of anhydrous ethanol (POCH) containing an internal standard (0.5 mL IS/10 mL EtOH) (Vitamins A and E in Serum/Plasma—HPLC, Chromsystems), vortexed and left for 10–15 min. Then, 400 µL MilliQ-grade deionized water (Merck Millipore) and 800 µL of hexane (Aldrich) were added to extract the vitamin. The samples were shaken vigorously for 2 h and centrifuged at 25,155×*g* for 10 min. Then, 400 µL of the upper layer (hexane) were collected, dried in Speed-Vac system (8 min), and dissolved in 100 µL of mobile phase (acetonitrile and methanol (Sigma-Aldrich), 80:20 (v/v), HPLC-grade). The samples were shaken vigorously overnight, centrifuged at 25,155×*g* for 10 min, and supernatants were injected into HPLC system. The method was validated with the reference material from Chromsystems.

#### Chromatography

The HPLC system consisted of 1525µ binary HPLC pump and 2707 autosampler, both from Waters. Retinol and α-tocopherol were quantified with Jasco FP-920 fluorimetric detector (Jasco Co.). The samples were separated in an isocratic system with a 5-µm Atlantis dC18 column equipped with guard cartridge (5 µm, 150 mm × 3.0, Waters). The mobile phase, consisting of acetonitrile methanol, 80:20 (v/v), was added at a flow rate of 1.5 mL/min, with 5-µL injection volume. The effluent was monitored by means of fluorimetric detection (λ_ex._ = 340 nm, λ_em._ = 472 nm for retinol, and λ_ex._ = 290 nm, λ_em._ = 330 nm for α-tocopherol and internal standard), and analyzed with Empower software.

### Gene expression analysis

Isolated leukocytes were stored at − 80 °C until the analysis. RNA was isolated with MagNA Pure 2.0 (Roche) following the procedures predicted by producent. Concentration, quality and integrity of total RNA aliquots were verified using standard procedures including spectrophotometrical methods and electrophoresis (details in Additional file 1). Purified RNA was stored at − 80 °C. The cDNA synthesis was performed using High-Capacity cDNA Reverse Transcription Kit (Applied Biosystems, catalog no. 43-688-14), according to the manufacturer’s instruction. The obtained cDNA was either used for qPCR setup immediately after obtaining, or stored at − 20 °C.

The RT-qPCR complies with the Minimum Information for Publication of Quantitative Real-time PCR Experiments (MIQE) guidelines. Three gene transcripts, *TET1, TET2* and *TET3*, were analyzed by relative quantitative RT-PCR (RT-qPCR) with relevant primers and probes labeled with fluorescein (FAM) from the Universal Probe Library (UPL, Roche) (see Table 1). Expressions of target genes were normalized for two selected reference genes, *HMBS* (GeneID: 3145) and *TBP* (GeneID: 6908), using UPL Ready Assay #100092149 and #100092158, respectively. Real-time PCR mixes (in 20-μL volumes) were prepared from cDNA following the standard procedures for LightCycler480 Probes Master (Roche), provided with the reagent set. The reactions were carried out according to qPCR Good Laboratory Practice (details in Additional file 1). The reaction for each gene was standardized against a standard curve, to estimate amplification efficiency, which was assessed based on a slope of the standard curve. Then, the samples were subjected to qPCR with measurement of *C*_t_, and amplification efficiencies were automatically calculated by LightCycler 480 software, version 1.5.1.62 (Roche). The same software was also used for sample setup, real-time PCR analysis and calculation of relative *C*_t_ values referred to as “Ratios”.

**Table 1:** Primers and short hydrolysis probes used for TETs mRNA expression analysis

Gene	Forward primer sequence	Reverse primer sequence	UPL	Probe sequence
*TET1*	5′-TCTGTTGTTGTGCCTCTGGA-3′	5′-GCCTTTAAAACTTTGGGCTTC-3′	#57	GGCCCCAG
*TET2*	5′-GCCTTTGCTCCTGTTGAGTT-3′	5′-ACAAGGCTGCCCTCTAGTTG-3′	#38	GGAAGCAG
*TET3*	5′-CACTCCGGAGAAGATCAAGC-3′	5′-GGACAATCCACCCTTCAGAG-3′	#1	CCTGGAGC
*TBP*	5′-GAACATCATGGATCAGAACAACA-3′	5′-ATAGGGATTCCGGGAGTCAT-3′	#87	CTGCCACC
*HMBS*	5′-TGCCCTGGAGAAGAATGAAG-3′	5′-CAGCATCATGAGGGTTTTCC-3′	#69	CTTCCTCC

### Statistical analysis

The results are presented as medians, interquartile ranges and non-outlier ranges. Normal distribution of the study variables was verified with Kolmogorov–Smirnov test with Lilliefors correction, and based on visual inspection of plotted histograms. Variables with normal distributions (5-mdC, retinol and α-tocopherol) were analyzed as “raw” data, while the variables with non-normal distributions (5-hmdC, 5-fdC, 5-cadC, 5-hmdU, 8-oxodG, ascorbate concentration and TET mRNA expression) were subjected to Box–Cox transformation prior to statistical analyses with parametric tests. One-way analysis of variance (ANOVA), LSD and Tukey post hoc tests were used to verify the significance of between-group differences. Associations between pairs of variables were analyzed based on Pearson correlation coefficients for raw or normalized data, where applicable. As recommended by Evans [24], Pearson’s correlations with r values of 0.20–0.39 were interpreted as weak, whereas those with r values of 0.40–0.59 and 0.60–0.79 as moderate and strong, respectively. To estimate the effects of ascorbate and retinol concentrations on DNA modification levels, the study subjects were divided into four subgroups (Q1–Q4), using the cut-off values corresponding to lower quartile, median and upper quartile in the controls. Multiple correlation (multiple R) coefficients were calculated for two independent variables (plasma ascorbate concentration and TETs mRNA expression), as the determinants of endogenous DNA modification contents in leukocytes. The relationships are presented as 3D-scatterplots with second-order polynomial function surface fitting. All statistical transformations and analyses were carried out with STATISTICA 13.1 PL [Dell Inc. (2016). Dell Statistica (data analysis software system), version 13. software.dell.com.]. The results were considered statistically significant at *p* values lower than 0.05.

## Results

### Distinct pattern of epigenetic DNA modification in leukocytes

**Fig. 1:**
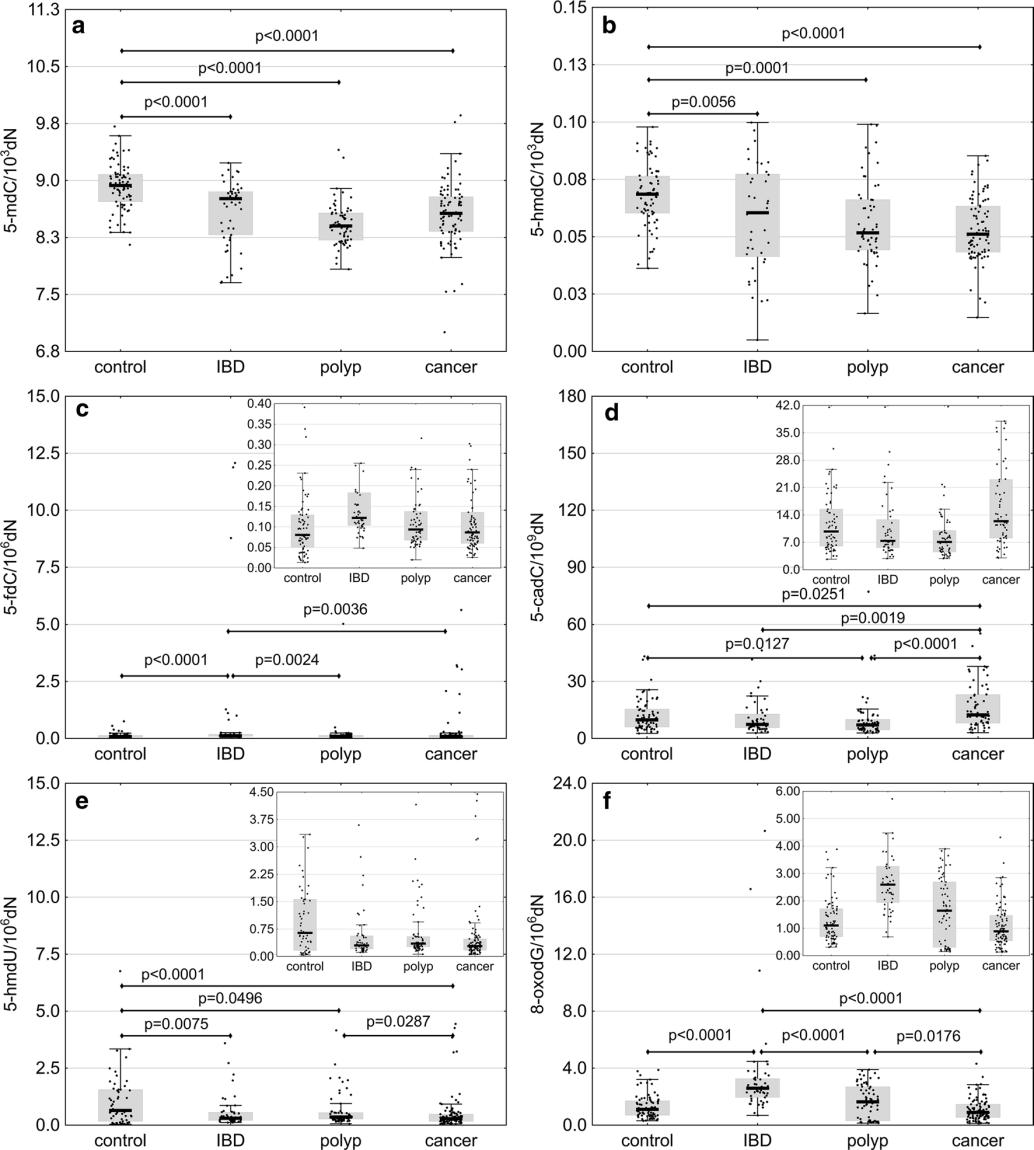
Levels of 5-mdC (**a**), 5-hmdC (**b**), 5-fdC (**c**), 5-cadC (**d**), 5-hmdU (**e**) and 8-oxodG (**f**) in leukocyte DNA from healthy controls (n = 79) and patients with inflammatory bowel disease (IBD, n = 51), adenomatous polyps (n = 67) and colorectal cancer (n = 136). The results presented as medians, interquartile ranges and non-outlier ranges. Raw (5-mdC) and normalized (other parameters) values were subjected to one-way analysis of variance (ANOVA) with LSD and Tukey post hoc tests

All patients, irrespective of their primary disease, presented with lower levels of 5-mdC (p < 0.0001) than the controls (Additional file 1: Table S2 and Fig. 1a). Also the levels of 5-hmdC in patients with IBD (p = 0.0056), polyps (p = 0.0001) and colorectal cancer (p < 0.0001) were lower than in healthy individuals (Additional file 1: Table S2 and Fig. 1b). Similar to 5-mdC and 5-hmdC concentrations, also the level of 5-hmdU turned out to be higher in healthy persons than in patients with IBD (p = 0.0075), polyps (p = 0.0496) and colorectal cancer (p < 0.0001) (Additional file 1: Table S2 and Fig. 1e). In the patient groups, the highest and the lowest levels of 5-hmdU were observed in individuals with polyps and colorectal cancer, respectively; these two groups differed significantly in terms of their 5-hmdU levels (p = 0.0287). Individuals with IBD presented with higher 5-fdC levels than persons with polyps (p = 0.0024), colorectal cancer patients (p = 0.0036) and healthy controls (p < 0.0001) (Fig. 1c and Additional file 1: Table S2). Also 8-oxodG level in persons with IBD turned out to be higher (1.6- to 2.9-fold) than in other study groups (p < 0.0001) (Fig. 1f and Additional file 1: Table S2). Individuals with polyps and colorectal cancer differed significantly in terms of their 8-oxodG levels (p = 0.0176). Finally, colorectal cancer patients presented with significantly higher 5-cadC levels than the controls (p = 0.0251), individuals with IBD (p = 0.0019) and persons with polyps (p < 0.0001) (Fig. 1d and Additional file 1: Table S2).

### Differences in plasma concentrations of ascorbate, retinol and α-tocopherol

**Fig. 2:**
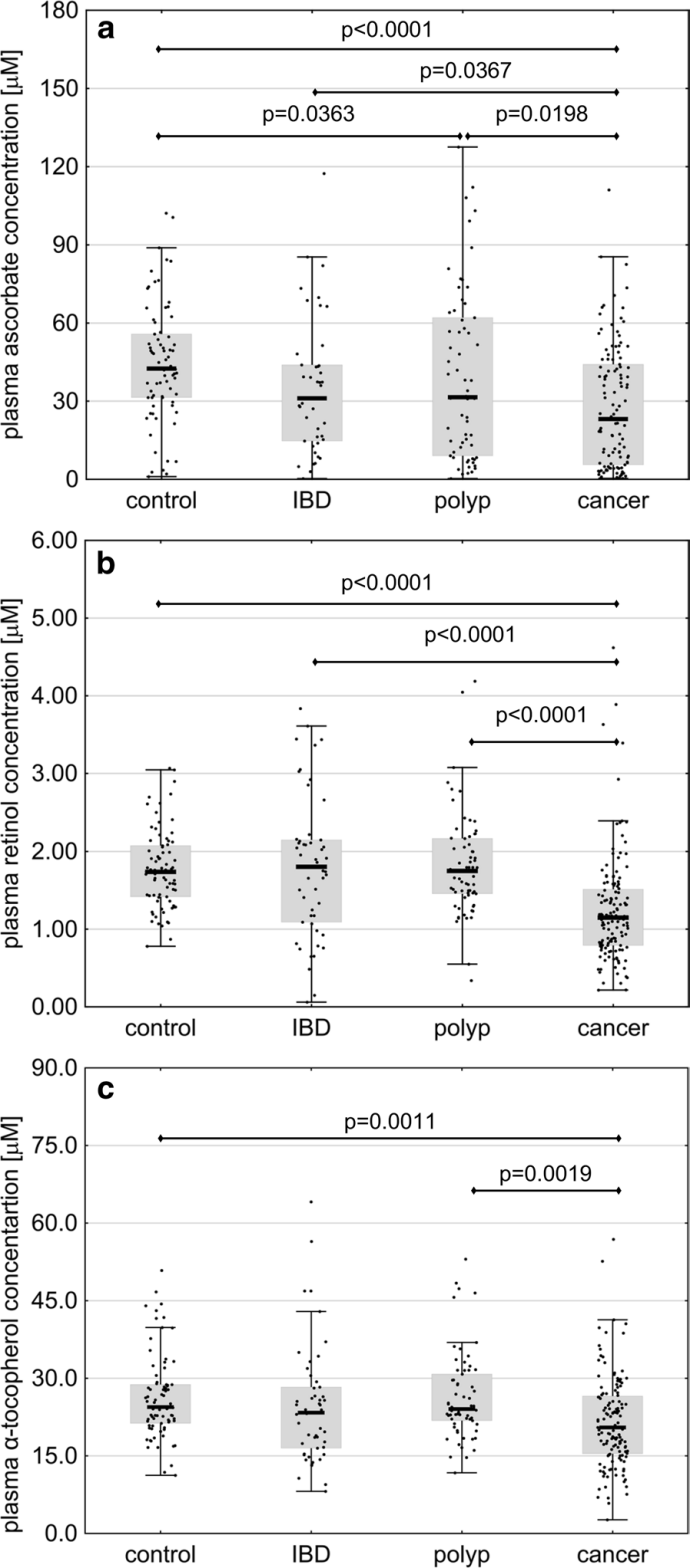
Plasma concentrations of ascorbate (**a**), retinol (**b**) and α-tocopherol (**c**) in healthy controls (n = 79) and patients with inflammatory bowel disease (IBD, n = 51), adenomatous polyps (n = 67) and colorectal cancer (n = 136). The results presented as medians, interquartile ranges and non-outlier ranges. Raw (retinol, α-tocopherol) or normalized (ascorbate) values were subjected to one-way analysis of variance (ANOVA) with LSD and Tukey post hoc tests

Plasma concentrations of ascorbate in colorectal cancer patients were lower than in the controls (p < 0.0001), individuals with IBD (p = 0.0367) and persons with polyps (p = 0.0198). Moreover, plasma concentrations of ascorbate in persons with polyps turned out to be lower than in the controls (p = 0.0363) (Fig. 2a and Additional file 1: Table S2). Colorectal cancer patients presented with significantly lower plasma concentrations of retinol than other study subjects (p < 0.0001) (Fig. 2b and Additional file 1: Table S2). The highest retinol levels were found in IBD patients. Plasma concentration of α-tocopherol, a vitamin with antioxidant properties, in colorectal cancer patients was lower than in the controls (p = 0.0011) and individuals with polyps (p = 0.0019) (Fig. 2c).

### Analysis of TET mRNA expressions in patients and healthy controls

**Fig. 3:**
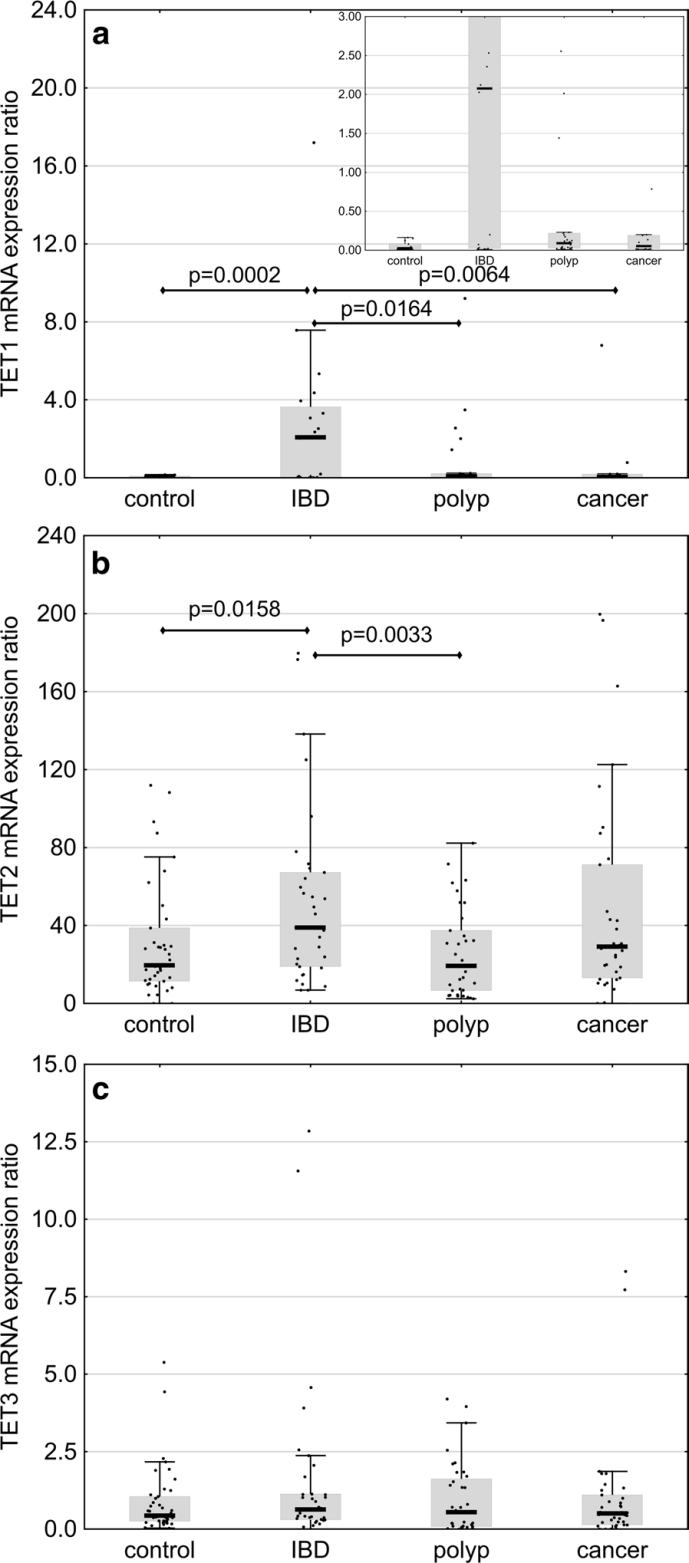
Expression of TET1 (**a**), TET2 (**b**) and TET3 (**c**) mRNA in healthy controls (n = 79) and patients with inflammatory bowel disease (IBD, n = 51), adenomatous polyps (n = 67) and colorectal cancer (n = 136). The results presented as medians, interquartile ranges and non-outlier ranges. Normalized values were subjected to one-way analysis of variance (ANOVA) with LSD and Tukey post hoc tests

Expression of TET1 in individuals with IBD turned out to be significantly higher than in the controls (p = 0.0002), persons with polyps (p = 0.0164) and colorectal cancer patients (p = 0.0064) (Fig. 3a and Additional file 1: Table S2). The latter three groups did not differ significantly in terms of their TET1 expressions. Moreover, statistically significant differences were found between the expressions of TET2 in IBD patients, controls (p = 0.0158) and persons with polyps (p = 0.0033) (Fig. 3b and Additional file 1: Table S2). Colorectal cancer patients did not differ from other study groups in terms of their TET2 expressions. Furthermore, no statistically significant between-group differences were observed in TET3 expressions (Fig. 3c and Additional file 1: Table S2).

### Mutual relationships between endogenous nucleobase modifications in leukocyte DNA

The level of 8-oxodG showed moderate positive correlation with the level of 5-fdC in patients with IBD (r = 0.5452, p < 0.0001), polyps (r = 0.5801, p < 0.0001) and colorectal cancer (r = 0.3954, p < 0.0001). Moreover, 8-oxodG correlated weakly with 5-cadC in IBD (r = 0.3141, p = 0.030) and colorectal cancer groups (r = 0.3909, p = 0.002), and with 5-hmdC in colorectal cancer patients (r = 0.2260, p = 0.044). A moderate positive correlation was observed between 8-oxodG and two epigenetic modifications, 5-cadC (= 0.4541, p < 0.0001) and 5-hmdC (r = 0.4418, p = 0.001) in patients with polyps. Furthermore, 8-oxodG correlated weakly with 5-hmdU in IBD (r = 0.3902, p = 0.006), polyp (r = 0.3718, p = 0.002) and colorectal cancer patients (r = 0.2425, p = 0.035) (Additional file 1: Figure S1). None of those correlations were observed in the controls. A weak positive substrate-product correlations were found between 5-fdC and its derivative, 5-cadC in healthy controls (r = 0.3434, p = 0.006) and colorectal cancer patients (r = 0.3124, p = 0.021). Moreover, 5-fdC correlated moderately with 5-cadC in the polyp group (r = 0.5361, p < 0.0001). A moderate inverse correlation was found between 5-mdC and 8-oxodG in IBD patients (r = − 0.4595, p = 0.001), along with a weak inverse correlation between 5-mdC and 5-fdC (r = −  0.3318, p = 0.020). Moreover, 5-mdC level in IBD patients correlated moderately with 5-hmdC content (r = 0.4248, p = 0.007). In the polyp group, 5-hmdU correlated strongly with 5-fdC (r = 0.6277, p < 0.0001), moderately with 5-hmdC (r = 0.4522, p = 0.001) and weakly with 5-cadC (r = 0.3344, p = 0.010). In colorectal cancer patients, the relationship between 5-hmdU and 5-fdC was moderate (r = 0.5631, p < 0.0001) and in the controls, weak (r = 0.3882, p = 0.004). Finally, weak albeit statistically significant correlations were found between 5-mdC and 5-cadC levels (r = 0.3118, p = 0.011), as well as between 5-hmdC and 5-cadC levels (r = 0.3229, p = 0.011) in healthy controls. A moderate correlation between 5-hmdC and 5-cadC levels was also observed in colorectal cancer patients (r = 0.4055, p = 0.002) (Additional file 1: Figures S2–S6).

### Correlational analysis of the role of ascorbate and TETs expression in the generation of endogenous nucleobase modifications in leukocyte DNA

**Fig. 4:**
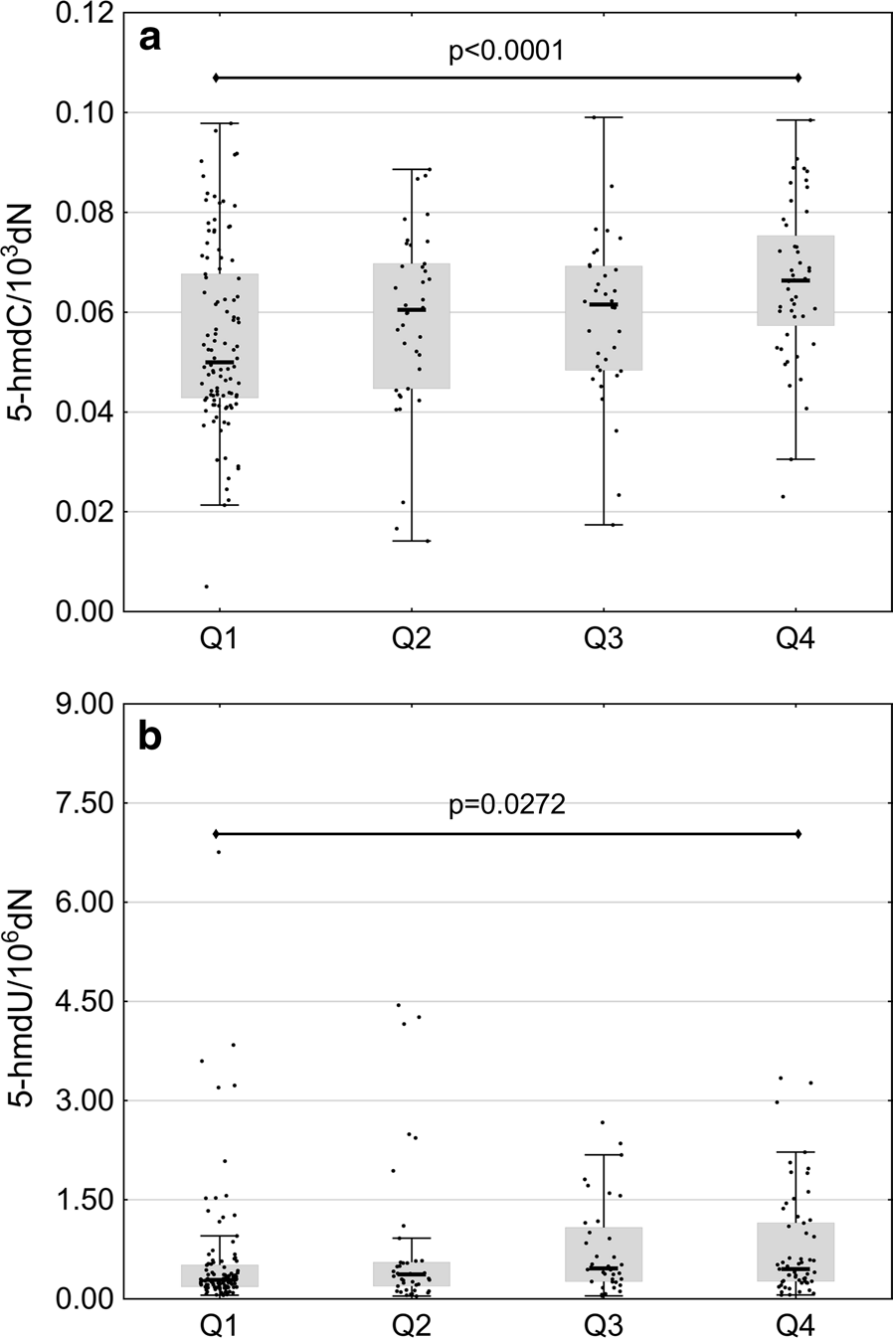
Selected, statistically significant associations between plasma concentration of ascorbate and the levels of 5-hmdC (**a**) and 5-hmdU (**b**). Complete correlation matrix is presented in Additional file 1: Figures S2–S6

In patients with polyps, weak albeit significant positive correlations were found between plasma concentrations of ascorbate and 5-hmdC (r = 0.3465, p = 0.013), 5-hmdU (r = 0.3009, p = 0.018) and 8-oxodG levels (r = 0.3722, p = 0.003) in leukocyte DNA. Persons whose plasma concentrations of ascorbate were below the lower and above the upper quartile for the controls differed significantly in terms of their 5-hmdC levels (p < 0.0001) (Fig. 4a). Plasma concentration of ascorbate in the controls correlated weakly with 5-hmdU level (r = 0.3807, p = 0.007).

While the positive correlation between TET2 and TET3 expressions in pooled groups was generally weak (r = 0.3876, p < 0.0001), it tended to be stronger in patients with polyps (r = 0.6803, p < 0.0001). In IBD patients, moderate inverse relationships were observed between TET2 expression and 5-fdC level (r = − 0.4413, p = 0.015), and between TET3 expression and 5-hmdC level (r = − 0.4506, p = 0.024). Moreover, 5-hmdU level in IBD group correlated weakly with TET2 (r = − 0.3844, p = 0.036) and TET3 expressions (r = − 0.3704, p = 0.037). Finally, moderate inverse correlations were found between TET1 expression and 5-hmdU level (r = − 0.4236, p = 0.035) in persons with polyps, and between TET2 expression and 5-cadC level (r = − 0.5324, p = 0.023) in colorectal cancer patients (Additional file 1: Figures S2–S6).

To provide a better insight into the role of ascorbate in epigenetic processes, we verified if its plasma concentration and TETs expression influenced the levels of endogenous modifications in leukocyte DNA. Plasma concentration of ascorbate and TET3 mRNA expression turned out to correlate positively with 5-hmdC (multiple R = 0.2407, p = 0.0462) and 5-hmdU levels (multiple R = 0.2665, p = 0.0112) (Additional file 1: Figure S7), whereas ascorbate concentration and TET2 mRNA expression showed positive correlations with 5-fdC (multiple R = 0.3034, p = 0.0043) and 5-hmdU contents (multiple R = 0.2413, p = 0.0404) (Additional file 1: Figure S8).

## Discussion

Although a molecular link between colon adenomas/IBD and carcinogenesis is yet to be established, it likely involves aberrant methylation and oxidative damage of DNA (reviewed [25, 26]), and those processes were postulated to precede colonic dysplasia and colorectal cancer development [27]. Furthermore, it cannot be excluded that aberrant methylation of DNA is somehow related to oxidative stress.

A growing body of evidence suggests that reduced content of 5-hmCyt may be characteristic not only for cancer tissue but also for precancerous lesions [11]. This in turn implies that this process may perpetuate during tumor progression.

Our present study showed for the first time that 5-hmCyt content in leukocytes decreased according to the following pattern: healthy controls > IBD patients > polyp patients > colorectal cancer patients (Fig. 1b). This suggests that a decrease in global level of 5-hmCyt observed during the course of colon cancer development is not limited solely to the malignant tissue, but may be also observed in surrogate tissues, such as leukocytes. This in turn implies that aberrant methylation of DNA may be a systemic process, rather than a local phenomenon. Aside from a decrease in 5-hmCyt content, leukocytes from all patient groups contained significantly less 5-hmUra than the cells from healthy controls (Fig. 1e).

According to Pfaffeneder et al. [8], 5-hmUra level undergoes changes during the course of epigenetic cell reprogramming, following the same pattern as other TET products, i.e. 5-hmCyt, 5-caCyt and 5-fCyt. This implies that 5-hmUra may have an epigenetic function, similar to other products of active DNA demethylation (reviewed in [28]). Our hereby presented results add to this evidence, suggesting that similar to 5-hmCyt, also 5-hmUra may be an epigenetic mark of carcinogenesis.

We demonstrated that the level of 5-fCyt, a higher-order oxidative epigenetic mark, was significantly higher in IBD patients than in other study groups (Fig. 1c). Interestingly, individuals with IBD presented also with significantly higher levels of 8-oxodG, an established marker of oxidative stress (Fig. 1f).

The association between inflammation and oxidative stress is well documented, and a number of previous studies demonstrated that inflammatory conditions and infections may be associated with an increase in 8-oxodG level. Inflammatory response may result in recruitment of activated leukocytes. This may lead to a “respiratory burst”, i.e. an increase in oxygen uptake with resultant enhanced release of reactive oxygen species (ROS), such as superoxide and hydrogen peroxide; this eventually contributes to oxidative stress and DNA damage (for review see [29]).

Noticeably, all patients participating in this study, irrespective of their underlying condition, showed statistically significant positive correlations between the leukocyte levels of all analyzed oxidized epigenetic modifications (except 5-hmdC in IBD group) and 8-oxodG content, while no such associations were found in healthy controls (Additional file 1: Figures S1, S3–S6).

Endogenous synthesis of free radicals probably does not constitute a principal reason behind the formation of epigenetic marks in cellular DNA [30]. Rather, environment characteristic for oxidative stress, linked with the pathogenesis, may influence factors responsible for the formation of the abovementioned modifications. Indeed, recent evidence suggests that oxidative stress may contribute to post-translational modulation of TET2 [31]. In line with those findings, we showed that the leukocyte contents of TET1 and TET2 mRNA in patients with IBD (a condition associated with more severe oxidative stress) were significantly higher than in other study groups (Fig. 3a, b). Furthermore, IBD patients presented with elevated levels of 5-fCyt. Since the structure of TET co-substrates (2-ketoglutarate, Fe^+2^) depends on redox state of the cell, altered activity of these enzymes may reflect the level of oxidative stress in IBD patients, i.e. the factor that might contribute to 5-fCyt formation. Moreover, it cannot be excluded that also superoxide (O^−2^), an anion radical of dioxygen and the precursor of free radicals, may play an important role in TET-mediated active DNA demethylation [32, 33].

The level of another higher-order oxidative modification of 5-mCyt, i.e. 5-caCyt, was the highest in leukocytes from colorectal cancer patients (Fig. 1d). Recent evidence suggests that TET2 may yield 5-fCyt and 5-caCyt without the release and dilution of 5-hmCyt, and consecutive steps of the iterative oxidation were postulated to be regulated by co-substrate levels [34]. Consequently, a persistent increase in oxidative stress may alter TET activity, promoting/down-regulating the generation of 5-fCyt and 5-caCyt during iterative oxidation of 5-mCyt. Taken altogether, this evidence suggests that the synthesis of epigenetic DNA modifications is linked to oxidative stress; however, this relationship seems to be complex and its exact character is still not completely understood.

In our present study, patients from all groups presented with significantly lower levels of 5-mCyt than the controls (Fig. 1a); the lowest 5-mCyt levels were observed in individuals with polyps and colorectal cancer. The distribution of 5-mCyt levels across the study groups followed a similar pattern as for 5-hmCyt: healthy controls > IBD patients > polyp and colorectal cancer patients. A dramatic decrease in 5-mCyt and 5-hmCyt levels may contribute to genomic instability, constituting a decisive step in colorectal cancer development. Interestingly, we found a significant inverse correlation between 5-mCyt and 8-oxodG levels in IBD patients (Additional file 1: Figures S1, S4), which constitutes another argument for a potential link between aberrant DNA methylation and oxidative stress.

A few previous studies demonstrated that ascorbate may enhance generation of 5-hmCyt in cultured cells, probably acting as a cofactor of TETs during the hydroxylation of 5-mCyt [14–17]. Recently, we have reported a spectacular increase in 5-hmUra level after stimulation with ascorbate [15]. In turn, our present study demonstrated a positive correlation between plasma concentration of ascorbate and the levels of two epigenetic modifications, 5-hmCyt and 5-hmUra in leukocyte DNA (Additional file 1: Figure S2). Moreover, we found a significant difference in the levels of these modifications in patients whose plasma concentrations of ascorbate were below the lower and above the upper quartile for the controls (Fig. 4a, b). It is of note that plasma concentrations of ascorbate may differ up to tenfold from person to person, and individuals in whom the level of this compound does not exceed the lower quartile were shown to be at increased risk of cancer mortality [35]. Previous studies demonstrated that if blood concentration of ascorbate remains at a physiological level (above 20 µM), leukocyte concentration of this compound reaches plateau, about 3 mM [36, 37]. Therefore, we analyzed the associations between DNA modifications in persons with higher ascorbate levels (above 40 µM) and in individuals with ascorbate concentrations below 20 µM (Additional file 1: Figure S9), in whom cellular uptake of this compound is impaired [36, 38]. Interestingly, participants from the former group presented with significantly higher levels of DNA modifications than the persons with ascorbate deficiency.

Probably, our study provided the first in vivo evidence for the involvement of ascorbate in the generation of epigenetic DNA modifications. Our hereby presented findings suggest that ascorbate may play a role in cancer control, preventing aberrant methylation of DNA.

To the best of our knowledge, this is the first study to show that each of the analyzed groups, healthy controls, individuals with IBD and adenomatous polyps and colorectal cancer patients, presented with a characteristic pattern of epigenetic modifications in their leukocyte DNA. Therefore, an important question arises about the mechanism(s) involved in the development of disease-specific epigenetic modification profiles. Perhaps, these were the consequences of oxidative stress (differences in redox status) associated with a given pathological condition, which contributed to the alterations of cellular metabolism and interfered with iterative-enzymatic DNA modification. In this context it is worth mentioning that our previous study documented presence of oxidative stress in leukocytes from patients with colorectal cancer and polyps [12, 13].

While the involvement of TETs in formation of all epigenetic modifications analyzed in this study raises no controversies, still little is known about the regulation of this process. Specifically, it is unclear why the oxidation of 5-mCyt either stops at 5-hmCyt stage or proceeds to 5-fCyt and 5-caCyt stages. One potential explanation is different affinity of TETs to 5-mCyt, 5-hmCyt and 5-fCyt (for review see [39, 40]). It is also possible that different proteins/factors recognize the modifications and determine their fate [41]. Interestingly, some recent experiments demonstrated that transcription factors, Myc and Max, and perhaps also a number of other regulatory proteins, may specifically recognize 5-caCyt, but have lesser affinity to 5-fCyt, and show only a trace of affinity to 5-mCyt and 5-hmCyt [42]. Moreover, a recent study conducted by Xiong et al. [43] showed that Sall4, an oncogenic protein which is overexpressed in colon cancer [44], may further enhance TET2-catalyzed oxidation of 5-hmCyt.

Recently, various isoforms of TETs were identified (reviewed in [45]), and it cannot be excluded that their activity may be tissue-specific. Moreover, miRNA may either upregulate or downregulate the expression of TETs mRNA [46].

All the factors mentioned above may contribute to different activity of TETs in patients with various pathological conditions, which in turn may result in the formation of disease-specific epigenetic modification patterns.

## Conclusions

To summarize, this study showed that environmental factors associated with some pathophysiological conditions linked to colorectal cancer development may alter the pattern of epigenetic modifications, and thus, may be involved in colorectal carcinogenesis. A relevant question is how colon function may be linked with the pattern of epigenetic modifications in peripheral leukocytes. As mentioned above, the level of these modifications may be directly associated with DNA demethylation/TETs activity which in turn may be influenced by ascorbate concentration [47]. Deficiency of ascorbate in the blood may be a marker of its shortage in other tissues, and indirectly manifest a disruption of DNA methylation-demethylation processes. Indeed, our recent study [15] demonstrated that physiological concentrations of ascorbate guarantee a stable level of 5-hmCyt, a modification which is necessary for epigenetic function of the cell. However, markedly higher concentrations of ascorbate were needed to obtain a sustained increase in 5-fCyt, 5-caCyt and 5-hmUra levels, and perhaps also to initiate the active demethylation process. The latter finding may reflect cell adaptation to altered environmental conditions.

## Additional file

**Additional file 1.** Tables S1–S3, Figures S1–S10, additional method.

## Abbreviations

TET: ten-eleven translocation
5-mCyt: 5-methylcytosine
5-hmCyt: 5-hydroxymethylcytosine
5-fCyt: 5-formylcytosine
5-caCyt: 5-carboxycytosine
5-hmUra: 5-hydroxymethyluracil
2D-UPLC-MS/MS: two-dimensional ultra-performance liquid chromatography with tandem mass spectrometry
8-oxodG: 8-oxo-7,8-dihydro-2′-deoxyguanosine
IBD: inflammatory bowel disease
5-mdC: 5-methyl-2′-deoxycytidine
5-hmdC: 5-(hydroxymethyl)-2′-deoxycytidine
5-fdC: 5-formyl-2′-deoxycytidine
5-cadC: 5-carboxy-2′-deoxycytidine
5-hmdU: 5-(hydroxymethyl)-2′-deoxyuridine
UPLC: ultra-performance liquid chromatography
ROS: reactive oxygen species
