# Supplementary material for: In vivo evidence of ascorbate involvement in the generation of epigenetic DNA modifications in leukocytes from patients with colorectal carcinoma, benign adenoma and inflammatory bowel disease
Source: J Transl Med. 2018 Jul 20;16:204. doi: 10.1186/s12967-018-1581-9 (PMC6053776; doi:10.1186/s12967-018-1581-9)
Supplement: Supplementary file 1 — Additional file 1. Tables S1–S3, Figures S1–S10, additional method. [file 12967_2018_1581_MOESM1_ESM.doc]

**Additional Information**

***In vivo* evidence of ascorbate involvement in the generation of epigenetic DNA modifications in leukocytes from patients with colorectal carcinoma, benign adenoma and inflammatory bowel disease**

Marta Starczak^1^, Ewelina Zarakowska^1^, Martyna Modrzejewska^1^, Tomasz Dziaman^1^, Anna Szpila^1^, Kinga Linowiecka^1^, Jolanta Guz^1^, Justyna Szpotan^1^, Maciej Gawronski^1^, Anna Labejszo^1^, Ariel Liebert^3^, Zbigniew Banaszkiewicz^2,4^, Maria Klopocka^3^, Marek Foksinski^1^, Daniel Gackowski^1*^, Ryszard Olinski^1*^

^1^ Department of Clinical Biochemistry, Faculty of Pharmacy, Collegium Medicum in Bydgoszcz, Nicolaus Copernicus University in Toruń, Karlowicza 24, 85-092 Bydgoszcz, Poland

^2^ Department of Vascular Surgery and Angiology, Faculty of Medicine, Collegium Medicum in Bydgoszcz, Nicolaus Copernicus University in Toruń, M. Curie Sklodowskiej 9, 85-094 Bydgoszcz, Poland

^3^ Department of Vascular Diseases and Internal Medicine, Faculty of Health Sciences, Collegium Medicum in Bydgoszcz, Nicolaus Copernicus University in Toruń, Ujejskiego 75, 85-168 Bydgoszcz, Poland

^4^ Department of General, Gastrointestinal, Colorectal and Oncological Surgery, Jan Biziel University No. 2 in Bydgoszcz, Ujejskiego 75, 85-168 Bydgoszcz, Poland

**Table of contents:**

Table. S1. Transition patterns specific detector settings and sources of standards for analysed compounds

Table. S2. Descriptive statistics for analyzed parameters in healthy controls and patients with inflammatory bowel disease (IBD), adenomatous polyps and colorectal cancer

Figure S1 Correlations of 8-oxodG and endogenous DNA modifications involved in methylation/demethylation pathways in leukocytes in three patients groups

Figure S2 Correlations in pooled groups

Figure S3 Correlations in control group

Figure S4 Correlations in IBD group

Figure S5 Correlations in polyp group

Figure S6 Correlations in cancer group

Figure S7 Multiple correlations between TET3 mRNA expression, plasma ascorbate concentration and levels of endogenous DNA modifications

Figure S8 Multiple correlations between TET2 mRNA expression, plasma ascorbate concentration and levels of endogenous DNA modifications

Figure S9 Selected, statistically significant associations between the levels of DNA modifications and plasma concentrations of ascorbate in individuals with concentration above 40 µM and in the group with concentration below 20µM.

Additional method - gene expression analysis

Table. S3 Alternative primers and short hydrolysis probes used for validation of previous TETs mRNA expression analysis

Figure S10 Correlations between different primers and short hydrolysis probes used for TETs mRNA expression analysis

| **compound name** |  | **relative response ratio** | **ionization mode** | **nominal molecular mass (Da)** | **pseudomolecular ion formulation** | **nominal parent ion (Da)** | **nominal daughter ion (Da)** | **capillary (kV)** | **cone (V)** | **collision (eV)** | **standard source** |
| --- | --- | --- | --- | --- | --- | --- | --- | --- | --- | --- | --- |
| 5-(hydroxymethyl)-2'-deoxycytidine | quantifier | 0.033 | ESI+ | 257 | [M+H]+ | 258 | 124 | 1.2 | 15 | 10 | Berry & Associates, Dexter, MI, USA |
|  | qualifier |  | ESI+ | 257 | [M+H]+ | 258 | 142 | 1.2 | 15 | 10 |  |
| [D_3_]-5-(hydroxymethyl)-2'-deoxycytidine | quantifier | 0.031 | ESI+ | 260 | [(M+3)+H]+ | 261 | 127 | 1.2 | 15 | 10 | Toronto Research Chemicals, Toronto, Canada |
|  | qualifier |  | ESI+ | 260 | [(M+3)+H]+ | 261 | 145 | 1.2 | 15 | 10 |  |
| 5-formyl-2'-deoxycytidine | quantifier | 0.182 | ESI- | 255 | [M-H]- | 254 | 121 | 3.5 | 28 | 18 | Berry & Associates, Dexter, MI, USA |
|  | qualifier |  | ESI- | 255 | [M-H]- | 254 | 138 | 3.5 | 28 | 18 |  |
| [^13^C_10_, ^15^N_2_]-5-formyl-2'-deoxycytidine | quantifier | 0.178 | ESI- | 267 | [(M+12)-H]- | 266 | 128 | 3.5 | 28 | 18 | own synthesis, see M&M section |
|  | qualifier |  | ESI- | 267 | [(M+12)-H]- | 266 | 145 | 3.5 | 28 | 18 |  |
| 5-carboxy-2'-deoxycytidine | quantifier | 0.318 | ESI- | 271 | [M-H]- | 270 | 110 | 3.5 | 20 | 20 | Berry & Associates, Dexter, MI, USA |
|  | qualifier |  | ESI- | 271 | [M-H]- | 270 | 93 | 3.5 | 20 | 20 |  |
| [^13^C_10_, ^15^N_2_]-5-carboxy-2'-deoxycytidine | quantifier | 0.300 | ESI- | 283 | [(M+12)-H]- | 282 | 116 | 3.5 | 20 | 20 | own synthesis, see M&M section |
|  | qualifier |  | ESI- | 283 | [(M+12)-H]- | 282 | 99 | 3.5 | 20 | 20 |  |
| 5-(hydroxymethyl)-2'-deoxyuridine | quantifier | 1.092 | ESI- | 258 | [M-H]- | 257 | 124 | 3.5 | 20 | 15 | Berry & Associates, Dexter, MI, USA |
|  | qualifier |  | ESI- | 258 | [M-H]- | 257 | 214 | 3.5 | 20 | 10 |  |
| [^13^C_10_, ^15^N_2_]-5-(hydroxymethyl)-2'-deoxyuridine | quantifier | 1.233 | ESI- | 270 | [(M+12)-H]- | 269 | 131 | 3.5 | 20 | 15 | own synthesis, see M&M section |
|  | qualifier |  | ESI- | 270 | [(M+12)-H]- | 269 | 224 | 3.5 | 20 | 10 |  |
| 8-oxo-2'-deoxyguanosine | quantifier | 0.280 | ESI+ | 283 | [M+H]+ | 284 | 168 | 1.2 | 20 | 15 | Sigma-Aldrich, St. Louis, MO, USA |
|  | qualifier |  | ESI+ | 283 | [M+H]+ | 284 | 140 | 1.2 | 20 | 30 |  |
| [^15^N_5_]-8-oxo-2'-deoxyguanosine | quantifier | 0.292 | ESI+ | 288 | [(M+5)+H]+ | 289 | 173 | 1.2 | 20 | 15 | Cambridge Isotope Laboratories, Tewksbury, MA, USA |
|  | qualifier |  | ESI+ | 288 | [(M+5)+H]+ | 289 | 145 | 1.2 | 20 | 30 |  |

Table. S1. Transition patterns specific detector settings and sources of standards for analysed compounds (relative response ratio=area under the peak of qualifier ion/ area under the peak of quantifier ion)

|  | control (n=79) | IBD (n=51) | polyp (n=67) | cancer (n=136) |
| --- | --- | --- | --- | --- |
|  | geometric mean  (min;max) | geometric mean  (min;max) | geometric mean  (min;max) | geometric mean  (min;max) |
|  | median (interquartile range) | median (interquartile range) | median (interquartile range) | median (interquartile range) |
| 5-methyl-2'-deoxycytidine  per 10^3^ dN | 8.914  (8.155;9.711) | 8.567  (7.655;9.232) | 8.422  (7.833;9.403) | 8.545  (7.005;9.858) |
|  | 8.934  (8.722-9.080) | 8.762  (8.286-8.853) | 8.401  (8.216-8.572) | 8.566  (8.300-8.786) |
| 5-(hydroxymethyl)-2'-deoxycytidine  per 10^3^ dN | 0.066  (0.036;0.098) | 0.052  (0.005;0.100) | 0.053  (0.017;0.099) | 0.051  (0.015;0.085) |
|  | 0.068  (0.060-0.076) | 0.060  (0.041-0.077) | 0.052  (0.044-0.066) | 0.051  (0.043-0.063) |
| 5-formyl-2'-deoxycytidine  per 10^6^ dN | 0.082  (0.013;0.758) | 0.190  (0.048;12.075) | 0.105  (0.020;5.028) | 0.115  (0.026;5.640) |
|  | 0.081  (0.051-0.129) | 0.122  (0.104-0.183) | 0.094  (0.068-0.137) | 0.087  (0.060-0.136) |
| 5-carboxy-2'-deoxycytidine  per 10^9^ dN | 10.031  (2.679;43.221) | 8.814  (2.882;46.340) | 7.606  (2.855;77.192) | 13.330  (2.998;55.269) |
|  | 9.779  (6.060-15.460) | 7.364  (5.661-12.809) | 7.086  (4.538-9.997) | 12.411  (8.084-23.029) |
| 5-(hydroxymethyl)-2’-deoxyuridine per 10^6^ dN | 0.546  (0.041;6.759) | 0.367  (0.114;3.599) | 0.425  (0.062;4.159) | 0.302  (0.055;4.444) |
|  | 0.643  (0.169-1.564) | 0.304  (0.208-0.563) | 0.353  (0.267-0.544) | 0.286  (0.171-0.482) |
| 8-oxo-7,8-dihydro-2'-deoxyguanosine  per 10^6^ dN | 1.089  (0.309;3.886) | 2.662  (0.688;20.659) | 1.065  (0.144;3.902) | 0.843  (0.131;4.321) |
|  | 1.103  (0.697-1.714) | 2.594  (1.948-3.258) | 1.641  (0.305-2.692) | 0.891  (0.545-1.466) |
| plasma ascorbate concentration [µM] | 35.698  (1.057;102.178) | 24.303  (0.345;117.367) | 23.568  (0.335;127.489) | 15.562  (0.383;111.076) |
|  | 42.525  (31.405-55.790) | 31.157  (14.761-43.942) | 31.533  (9.071-62.059) | 23.213  (5.664-44.138) |
| plasma retinol concentration [µM] | 1.703  (0.780;3.067) | 1.493  (0.062;3.835) | 1.704  (0.339;4.189) | 1.084  (0.217;4.620) |
|  | 1.739  (1.418-2.074) | 1.803  (1.091-2.150) | 1.749  (1.457-2.169) | 1.150  (0.793-1.513) |
| plasma α-tocopherol concentration [µM] | 25.143  (11.260;50.832) | 22.432  (8.156;64.118) | 25.227  (11.744;53.036) | 19.684  (2.656;56.867) |
|  | 24.437  (21.320-28.777) | 23.344  (16.509-28.305) | 24.044  (21.844-30.819) | 20.521  (15.436-26.546) |
| TET1 mRNA expression ratio | 0.031  (0.010;0.165) | 0.414  (0.010;17.200) | 0.108  (0.010;9.203) | 0.070  (0.013;6.798) |
|  | 0.027  (0.013-0.081) | 2.076  (0.024-3.632) | 0.090  (0.028-0.218) | 0.054  (0.018-0.190) |
| TET2 mRNA expression ratio | 7.629  (0.000;112.000) | 36.386  (6.813;179.700) | 16.042  (2.489;82.270) | 15.916  (0.000;199.700) |
|  | 19.610  (11.470-38.750) | 38.920  (18.900-67.220) | 19.315  (6.660-37.520) | 29.150  (13.120-71.250) |
| TET3 mRNA expression ratio | 0.500  (0.031;5.385) | 0.437  (0.000;12.850) | 0.329  (0.002;4.204) | 0.094  (0.000;38.230) |
|  | 0.435  (0.258-1.051) | 0.634  (0.304-1.131) | 0.547  (0.090-1.619) | 0.509  (0.143-1.103) |

Table. S2. Descriptive statistics for analyzed parameters in healthy controls and patients with inflammatory bowel disease (IBD), adenomatous polyps and colorectal cancer


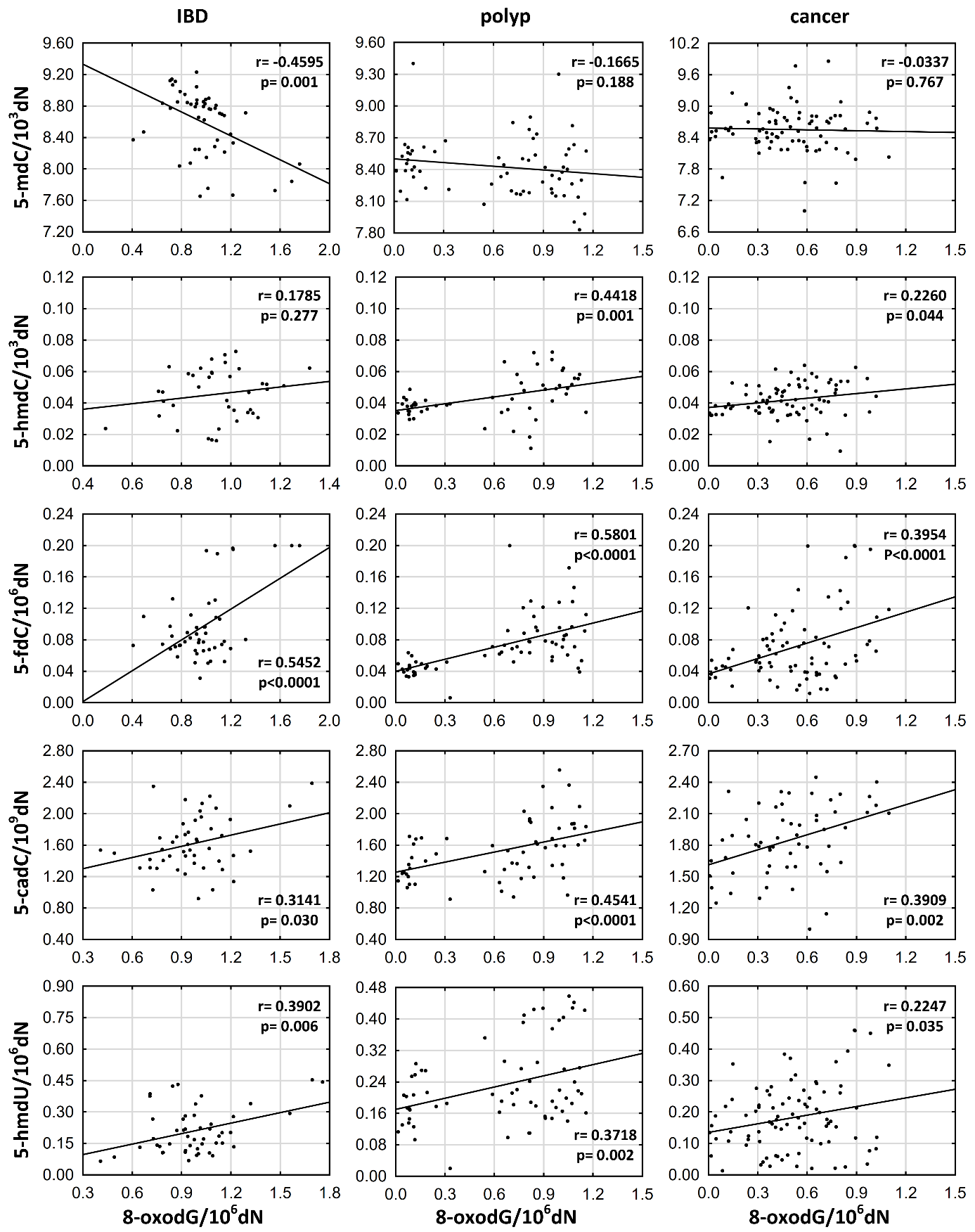


Figure S1 Correlations of 8-oxodG and endogenous DNA modifications involved in methylation/demethylation pathways in leukocytes in three patients groups: inflammatory bowel disease (IBD, n=51), adenomatous polyps (n=67) and colorectal cancer (n=136). No significant correlations has been found in control group.


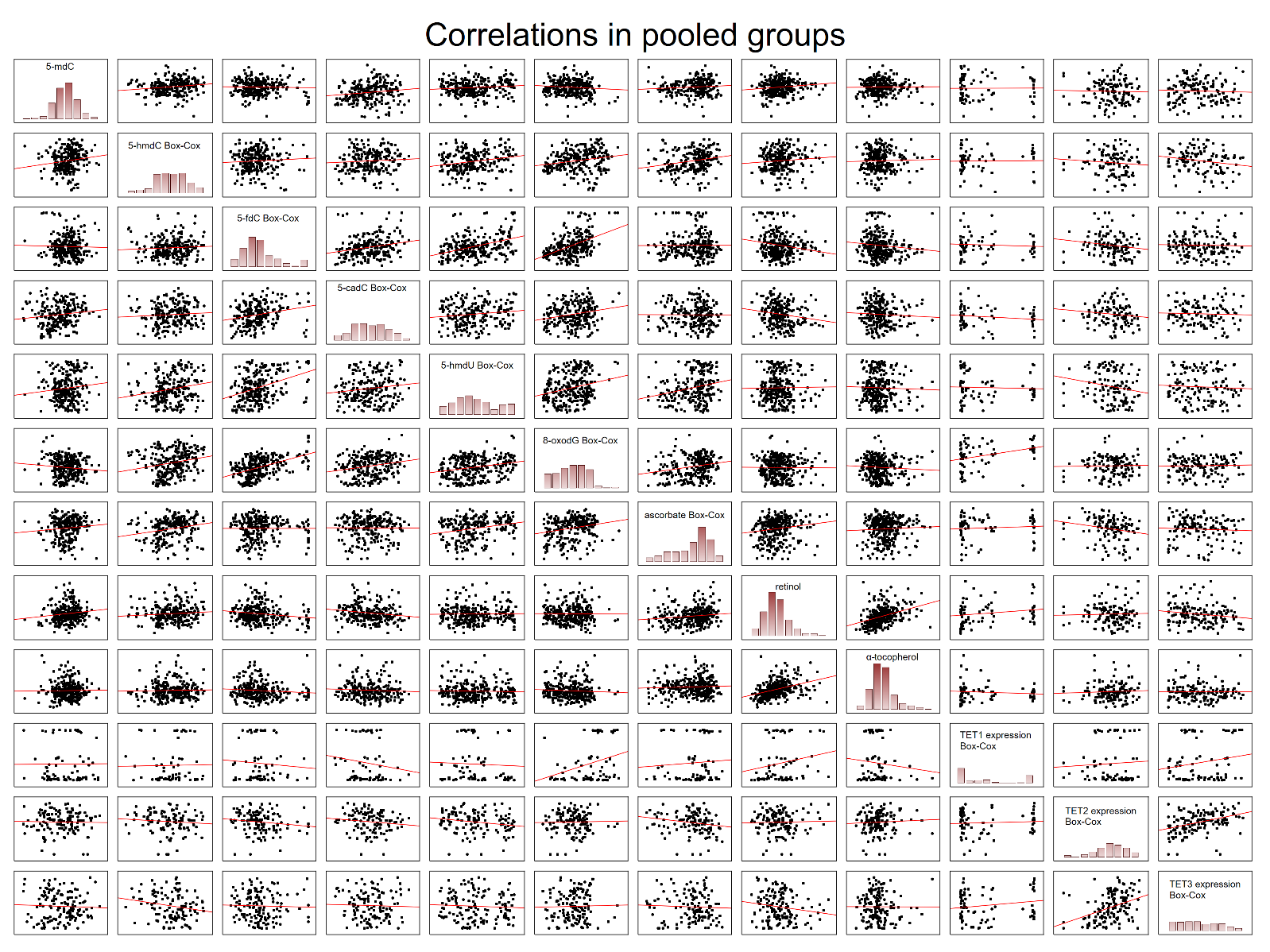


|  | 5-mdC | 5-hmdC Box-Cox | 5-fdC Box-Cox | 5-cadC Box-Cox | 5-hmdU Box-Cox | 8-oxodG Box-Cox | ascorbate Box-Cox | retinol | TET1 expression Box-Cox | TET2 expression Box-Cox | TET3 expression Box-Cox |
| --- | --- | --- | --- | --- | --- | --- | --- | --- | --- | --- | --- |
| 5-mdC | 1.0000 | .1537 | -.0229 | .1366 | .1066 | -.0956 | .0923 | .1354 | .0046 | -.0224 | -.0415 |
|  | N=286 | N=254 | N=271 | N=234 | N=259 | N=280 | N=247 | N=279 | N=72 | N=134 | N=143 |
|  | p= --- | p=.014 | p=.707 | p=.037 | p=.087 | p=.111 | p=.148 | p=.024 | p=.970 | p=.797 | p=.623 |
| 5-hmdC Box-Cox | .1537 | 1.0000 | .0625 | .0690 | .2111 | .2144 | .2149 | .0854 | .0146 | -.0927 | -.1926 |
|  | N=254 | N=257 | N=243 | N=209 | N=231 | N=252 | N=221 | N=250 | N=61 | N=112 | N=119 |
|  | p=.014 | p= --- | p=.332 | p=.321 | p=.001 | p=.001 | p=.001 | p=.178 | p=.911 | p=.331 | p=.036 |
| 5-fdC Box-Cox | -.0229 | .0625 | 1.0000 | .2246 | .3822 | .4723 | .0049 | -.1690 | -.0718 | -.1473 | -.0289 |
|  | N=271 | N=243 | N=284 | N=231 | N=255 | N=276 | N=247 | N=277 | N=69 | N=126 | N=135 |
|  | p=.707 | p=.332 | p= --- | p=.001 | p=.000 | p=.000 | p=.939 | p=.005 | p=.558 | p=.100 | p=.740 |
| 5-cadC Box-Cox | .1366 | .0690 | .2246 | 1.0000 | .1351 | .2207 | -.0038 | -.1698 | -.1479 | -.1313 | -.0353 |
|  | N=234 | N=209 | N=231 | N=238 | N=223 | N=237 | N=204 | N=234 | N=62 | N=115 | N=121 |
|  | p=.037 | p=.321 | p=.001 | p= --- | p=.044 | p=.001 | p=.957 | p=.009 | p=.251 | p=.162 | p=.700 |
| 5-hmdU Box-Cox | .1066 | .2111 | .3822 | .1351 | 1.0000 | .2444 | .2448 | .0189 | -.0438 | -.1931 | -.0494 |
|  | N=259 | N=231 | N=255 | N=223 | N=270 | N=265 | N=233 | N=264 | N=72 | N=132 | N=141 |
|  | p=.087 | p=.001 | p=.000 | p=.044 | p= --- | p=.000 | p=.000 | p=.760 | p=.715 | p=.027 | p=.561 |
| 8-oxodG Box-Cox | -.0956 | .2144 | .4723 | .2207 | .2444 | 1.0000 | .2205 | -.0072 | .3289 | .0236 | .0244 |
|  | N=280 | N=252 | N=276 | N=237 | N=265 | N=293 | N=254 | N=287 | N=72 | N=134 | N=143 |
|  | p=.111 | p=.001 | p=.000 | p=.001 | p=.000 | p= --- | p=.000 | p=.903 | p=.005 | p=.786 | p=.772 |
| ascorbate Box-Cox | .0923 | .2149 | .0049 | -.0038 | .2448 | .2205 | 1.0000 | .1434 | .0712 | -.1906 | -.0456 |
|  | N=247 | N=221 | N=247 | N=204 | N=233 | N=254 | N=308 | N=306 | N=71 | N=124 | N=136 |
|  | p=.148 | p=.001 | p=.939 | p=.957 | p=.000 | p=.000 | p= --- | p=.012 | p=.555 | p=.034 | p=.598 |
| retinol | .1354 | .0854 | -.1690 | -.1698 | .0189 | -.0072 | .1434 | 1.0000 | .1895 | .0308 | -.1615 |
|  | N=279 | N=250 | N=277 | N=234 | N=264 | N=287 | N=306 | N=348 | N=75 | N=139 | N=150 |
|  | p=.024 | p=.178 | p=.005 | p=.009 | p=.760 | p=.903 | p=.012 | p= --- | p=.103 | p=.719 | p=.048 |
| α-tocopherol | .0162 | .0284 | -.0991 | -.0582 | -.0329 | -.0655 | .0485 | .3730 | 1.0000 | -.1045 | .0554 |
|  | N=279 | N=250 | N=277 | N=234 | N=264 | N=287 | N=306 | N=348 | N=348 | N=75 | N=139 |
|  | p=.788 | p=.654 | p=.100 | p=.376 | p=.594 | p=.269 | p=.398 | p=.000 | p= --- | p=.372 | p=.517 |
| TET1 expression Box-Cox | .0046 | .0146 | -.0718 | -.1479 | -.0438 | .3289 | .0712 | .1895 | 1.0000 | .0568 | .1760 |
|  | N=72 | N=61 | N=69 | N=62 | N=72 | N=72 | N=71 | N=75 | N=79 | N=71 | N=79 |
|  | p=.970 | p=.911 | p=.558 | p=.251 | p=.715 | p=.005 | p=.555 | p=.103 | p= --- | p=.638 | p=.121 |
| TET2 expression Box-Cox | -.0224 | -.0927 | -.1473 | -.1313 | -.1931 | .0236 | -.1906 | .0308 | .0568 | 1.0000 | .3876 |
|  | N=134 | N=112 | N=126 | N=115 | N=132 | N=134 | N=124 | N=139 | N=71 | N=144 | N=144 |
|  | p=.797 | p=.331 | p=.100 | p=.162 | p=.027 | p=.786 | p=.034 | p=.719 | p=.638 | p= --- | p=.000 |
| TET3 expression Box-Cox | -.0415 | -.1926 | -.0289 | -.0353 | -.0494 | .0244 | -.0456 | -.1615 | .1760 | .3876 | 1.0000 |
|  | N=143 | N=119 | N=135 | N=121 | N=141 | N=143 | N=136 | N=150 | N=79 | N=144 | N=156 |
|  | p=.623 | p=.036 | p=.740 | p=.700 | p=.561 | p=.772 | p=.598 | p=.048 | p=.121 | p=.000 | p= --- |

Figure S2 Correlations in pooled groups


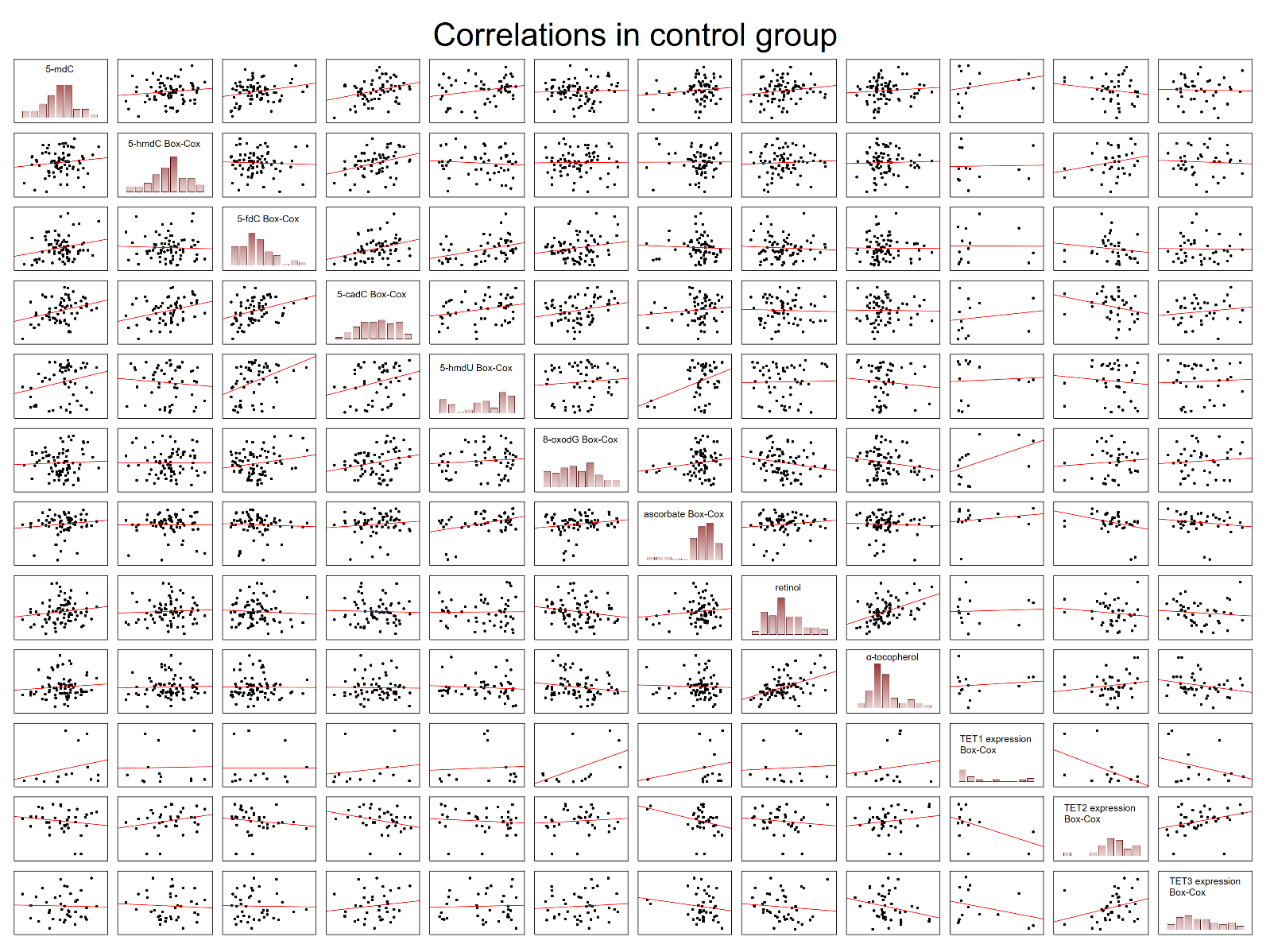


|  | 5-mdC | 5-hmdC Box-Cox | 5-fdC Box-Cox | 5-cadC Box-Cox | 5-hmdU Box-Cox | 8-oxodG Box-Cox | ascorbate Box-Cox | retinol | TET1 expression Box-Cox | TET2 expression Box-Cox | TET3 expression Box-Cox |
| --- | --- | --- | --- | --- | --- | --- | --- | --- | --- | --- | --- |
| 5-mdC | 1.0000 | .1295 | .2373 | .3118 | .2504 | .0449 | .1280 | .1540 | .2593 | -.1581 | -.0309 |
|  | N=79 | N=75 | N=76 | N=65 | N=54 | N=78 | N=71 | N=76 | N=14 | N=37 | N=40 |
|  | p= --- | p=.268 | p=.039 | p=.011 | p=.068 | p=.696 | p=.287 | p=.184 | p=.371 | p=.350 | p=.850 |
| 5-hmdC Box-Cox | .1295 | 1.0000 | -.0379 | .3229 | -.0936 | .0095 | .0023 | .0544 | .0223 | .2375 | -.0595 |
|  | N=75 | N=75 | N=72 | N=61 | N=50 | N=74 | N=67 | N=72 | N=13 | N=35 | N=38 |
|  | p=.268 | p= --- | p=.752 | p=.011 | p=.518 | p=.936 | p=.985 | p=.650 | p=.942 | p=.169 | p=.723 |
| 5-fdC Box-Cox | .2373 | -.0379 | 1.0000 | .3434 | .3882 | .1985 | -.0552 | -.0649 | -.0014 | -.1412 | -.0163 |
|  | N=76 | N=72 | N=76 | N=63 | N=52 | N=75 | N=68 | N=73 | N=14 | N=36 | N=39 |
|  | p=.039 | p=.752 | p= --- | p=.006 | p=.004 | p=.088 | p=.655 | p=.585 | p=.996 | p=.412 | p=.922 |
| 5-cadC Box-Cox | .3118 | .3229 | .3434 | 1.0000 | .2707 | .2476 | .1137 | -.0327 | .1513 | -.2757 | .1462 |
|  | N=65 | N=61 | N=63 | N=65 | N=50 | N=65 | N=58 | N=63 | N=13 | N=35 | N=37 |
|  | p=.011 | p=.011 | p=.006 | p= --- | p=.057 | p=.047 | p=.396 | p=.799 | p=.622 | p=.109 | p=.388 |
| 5-hmdU Box-Cox | .2504 | -.0936 | .3882 | .2707 | 1.0000 | .0923 | .3807 | .0177 | .0630 | -.0929 | .0409 |
|  | N=54 | N=50 | N=52 | N=50 | N=54 | N=54 | N=49 | N=52 | N=14 | N=35 | N=38 |
|  | p=.068 | p=.518 | p=.004 | p=.057 | p= --- | p=.507 | p=.007 | p=.901 | p=.831 | p=.595 | p=.808 |
| 8-oxodG Box-Cox | .0449 | .0095 | .1985 | .2476 | .0923 | 1.0000 | .1679 | -.1966 | .5155 | .0942 | .0823 |
|  | N=78 | N=74 | N=75 | N=65 | N=54 | N=78 | N=70 | N=75 | N=14 | N=37 | N=40 |
|  | p=.696 | p=.936 | p=.088 | p=.047 | p=.507 | p= --- | p=.165 | p=.091 | p=.059 | p=.579 | p=.614 |
| ascorbate Box-Cox | .1280 | .0023 | -.0552 | .1137 | .3807 | .1679 | 1.0000 | .1258 | .1925 | -.3207 | -.1636 |
|  | N=71 | N=67 | N=68 | N=58 | N=49 | N=70 | N=73 | N=72 | N=15 | N=37 | N=41 |
|  | p=.287 | p=.985 | p=.655 | p=.396 | p=.007 | p=.165 | p= --- | p=.292 | p=.492 | p=.053 | p=.307 |
| Re tinol | .1540 | .0544 | -.0649 | -.0327 | .0177 | -.1966 | .1258 | 1.0000 | .0526 | -.1285 | -.1013 |
|  | N=76 | N=72 | N=73 | N=63 | N=52 | N=75 | N=72 | N=78 | N=14 | N=39 | N=42 |
|  | p=.184 | p=.650 | p=.585 | p=.799 | p=.901 | p=.091 | p=.292 | p= --- | p=.858 | p=.435 | p=.523 |
| α-tocopherol | .0910 | .0341 | -.0092 | -.0182 | -.0925 | -.1752 | -.0405 | .4627 | 1.0000 | .1358 | .1606 |
|  | N=76 | N=72 | N=73 | N=63 | N=52 | N=75 | N=72 | N=78 | N=78 | N=14 | N=39 |
|  | p=.434 | p=.776 | p=.938 | p=.887 | p=.514 | p=.133 | p=.736 | p=.000 | p= --- | p=.643 | p=.329 |
| TET1 expression Box-Cox | .2593 | .0223 | -.0014 | .1513 | .0630 | .5155 | .1925 | .0526 | 1.0000 | -.5165 | -.3079 |
|  | N=14 | N=13 | N=14 | N=13 | N=14 | N=14 | N=15 | N=14 | N=15 | N=13 | N=15 |
|  | p=.371 | p=.942 | p=.996 | p=.622 | p=.831 | p=.059 | p=.492 | p=.858 | p= --- | p=.071 | p=.264 |
| TET2 expression Box-Cox | -.1581 | .2375 | -.1412 | -.2757 | -.0929 | .0942 | -.3207 | -.1285 | -.5165 | 1.0000 | .3133 |
|  | N=37 | N=35 | N=36 | N=35 | N=35 | N=37 | N=37 | N=39 | N=13 | N=39 | N=39 |
|  | p=.350 | p=.169 | p=.412 | p=.109 | p=.595 | p=.579 | p=.053 | p=.435 | p=.071 | p= --- | p=.052 |
| TET3 expression Box-Cox | -.0309 | -.0595 | -.0163 | .1462 | .0409 | .0823 | -.1636 | -.1013 | -.3079 | .3133 | 1.0000 |
|  | N=40 | N=38 | N=39 | N=37 | N=38 | N=40 | N=41 | N=42 | N=15 | N=39 | N=43 |
|  | p=.850 | p=.723 | p=.922 | p=.388 | p=.808 | p=.614 | p=.307 | p=.523 | p=.264 | p=.052 | p= --- |

Figure S3 Correlations in control group


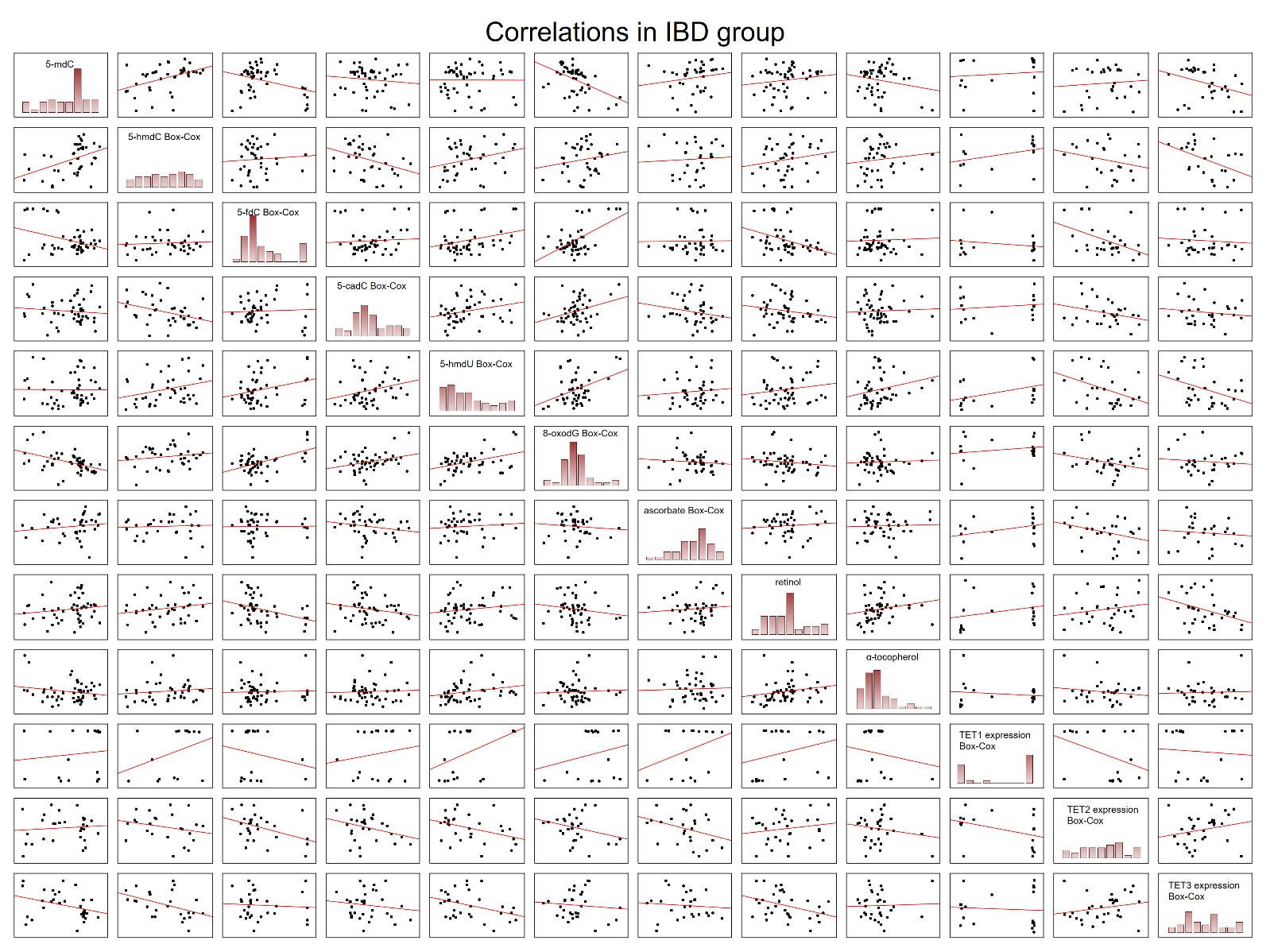


|  | 5-mdC | 5-hmdC Box-Cox | 5-fdC Box-Cox | 5-cadC Box-Cox | 5-hmdU Box-Cox | 8-oxodG Box-Cox | ascorbate Box-Cox | retinol | TET1 expression Box-Cox | TET2 expression Box-Cox | TET3 expression Box-Cox |
| --- | --- | --- | --- | --- | --- | --- | --- | --- | --- | --- | --- |
| 5-mdC | 1.0000 | .4248 | -.3318 | -.1060 | -.0061 | -.4595 | .1585 | .1473 | .1067 | .0870 | -.3333 |
|  | N=49 | N=39 | N=49 | N=48 | N=49 | N=49 | N=40 | N=49 | N=18 | N=30 | N=32 |
|  | p= --- | p=.007 | p=.020 | p=.473 | p=.967 | p=.001 | p=.329 | p=.312 | p=.673 | p=.648 | p=.062 |
| 5-hmdC Box-Cox | .4248 | 1.0000 | .0650 | -.3510 | .2828 | .1785 | .0533 | .1927 | .3465 | -.2398 | -.4506 |
|  | N=39 | N=39 | N=39 | N=39 | N=39 | N=39 | N=31 | N=39 | N=15 | N=24 | N=25 |
|  | p=.007 | p= --- | p=.694 | p=.028 | p=.081 | p=.277 | p=.776 | p=.240 | p=.206 | p=.259 | p=.024 |
| 5-fdC Box-Cox | -.3318 | .0650 | 1.0000 | .0549 | .2767 | .5452 | .0108 | -.3697 | -.1923 | -.4413 | -.0718 |
|  | N=49 | N=39 | N=49 | N=48 | N=49 | N=49 | N=40 | N=49 | N=18 | N=30 | N=32 |
|  | p=.020 | p=.694 | p= --- | p=.711 | p=.054 | p=.000 | p=.947 | p=.009 | p=.445 | p=.015 | p=.696 |
| 5-cadC Box-Cox | -.1060 | -.3510 | .0549 | 1.0000 | .2703 | .3141 | -.1994 | -.1943 | .1490 | -.2869 | -.1381 |
|  | N=48 | N=39 | N=48 | N=48 | N=48 | N=48 | N=40 | N=48 | N=18 | N=29 | N=31 |
|  | p=.473 | p=.028 | p=.711 | p= --- | p=.063 | p=.030 | p=.217 | p=.186 | p=.555 | p=.131 | p=.459 |
| 5-hmdU Box-Cox | -.0061 | .2828 | .2767 | .2703 | 1.0000 | .3902 | .0945 | .1542 | .3962 | -.3844 | -.3704 |
|  | N=49 | N=39 | N=49 | N=48 | N=49 | N=49 | N=40 | N=49 | N=18 | N=30 | N=32 |
|  | p=.967 | p=.081 | p=.054 | p=.063 | p= --- | p=.006 | p=.562 | p=.290 | p=.104 | p=.036 | p=.037 |
| 8-oxodG Box-Cox | -.4595 | .1785 | .5452 | .3141 | .3902 | 1.0000 | -.0924 | -.1513 | .2055 | -.2820 | -.1011 |
|  | N=49 | N=39 | N=49 | N=48 | N=49 | N=49 | N=40 | N=49 | N=18 | N=30 | N=32 |
|  | p=.001 | p=.277 | p=.000 | p=.030 | p=.006 | p= --- | p=.571 | p=.299 | p=.413 | p=.131 | p=.582 |
| ascorbate Box-Cox | .1585 | .0533 | .0108 | -.1994 | .0945 | -.0924 | 1.0000 | .0938 | .3356 | -.3318 | -.0884 |
|  | N=40 | N=31 | N=40 | N=40 | N=40 | N=40 | N=43 | N=42 | N=17 | N=25 | N=27 |
|  | p=.329 | p=.776 | p=.947 | p=.217 | p=.562 | p=.571 | p= --- | p=.555 | p=.188 | p=.105 | p=.661 |
| retinol | .1473 | .1927 | -.3697 | -.1943 | .1542 | -.1513 | .0938 | 1.0000 | .2582 | .1739 | -.3676 |
|  | N=49 | N=39 | N=49 | N=48 | N=49 | N=49 | N=42 | N=51 | N=19 | N=31 | N=33 |
|  | p=.312 | p=.240 | p=.009 | p=.186 | p=.290 | p=.299 | p=.555 | p= --- | p=.286 | p=.349 | p=.035 |
| α-tocopherol | -.1918 | .1264 | .0349 | .0472 | .2348 | .0482 | .0367 | .1987 | 1.0000 | -.1519 | -.1661 |
|  | N=49 | N=39 | N=49 | N=48 | N=49 | N=49 | N=42 | N=51 | N=51 | N=19 | N=31 |
|  | p=.187 | p=.443 | p=.812 | p=.750 | p=.104 | p=.742 | p=.817 | p=.162 | p= --- | p=.535 | p=.372 |
| TET1 expression Box-Cox | .1067 | .3465 | -.1923 | .1490 | .3962 | .2055 | .3356 | .2582 | 1.0000 | -.3835 | -.0726 |
|  | N=18 | N=15 | N=18 | N=18 | N=18 | N=18 | N=17 | N=19 | N=20 | N=18 | N=20 |
|  | p=.673 | p=.206 | p=.445 | p=.555 | p=.104 | p=.413 | p=.188 | p=.286 | p= --- | p=.116 | p=.761 |
| TET2 expression Box-Cox | .0870 | -.2398 | -.4413 | -.2869 | -.3844 | -.2820 | -.3318 | .1739 | -.3835 | 1.0000 | .2131 |
|  | N=30 | N=24 | N=30 | N=29 | N=30 | N=30 | N=25 | N=31 | N=18 | N=33 | N=33 |
|  | p=.648 | p=.259 | p=.015 | p=.131 | p=.036 | p=.131 | p=.105 | p=.349 | p=.116 | p= --- | p=.234 |
| TET3 expression Box-Cox | -.3333 | -.4506 | -.0718 | -.1381 | -.3704 | -.1011 | -.0884 | -.3676 | -.0726 | .2131 | 1.0000 |
|  | N=32 | N=25 | N=32 | N=31 | N=32 | N=32 | N=27 | N=33 | N=20 | N=33 | N=35 |
|  | p=.062 | p=.024 | p=.696 | p=.459 | p=.037 | p=.582 | p=.661 | p=.035 | p=.761 | p=.234 | p= --- |

Figure S4 Correlations in IBD group


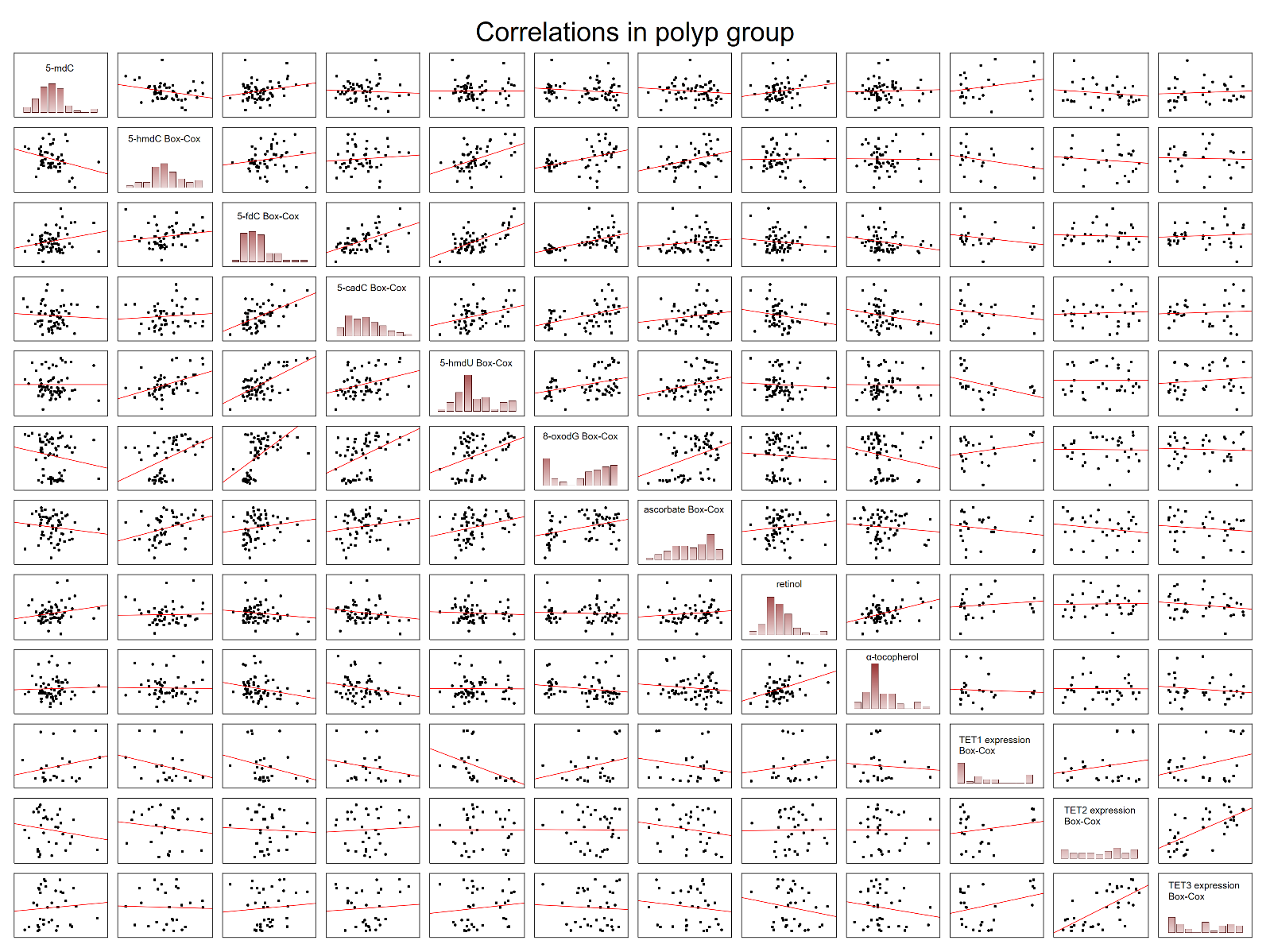


|  | 5-mdC | 5-hmdC Box-Cox | 5-fdC Box-Cox | 5-cadC Box-Cox | 5-hmdU Box-Cox | 8-oxodG Box-Cox | ascorbate Box-Cox | retinol | TET1 expression Box-Cox | TET2 expression Box-Cox | TET3 expression Box-Cox |
| --- | --- | --- | --- | --- | --- | --- | --- | --- | --- | --- | --- |
| 5-mdC | 1.0000 | -.2861 | .2368 | -.0618 | -.0001 | -.1665 | -.1314 | .2069 | .2373 | -.1587 | .0821 |
|  | N=64 | N=54 | N=64 | N=58 | N=64 | N=64 | N=60 | N=64 | N=25 | N=33 | N=35 |
|  | p= --- | p=.036 | p=.060 | p=.645 | p=.999 | p=.188 | p=.317 | p=.101 | p=.253 | p=.378 | p=.639 |
| 5-hmdC Box-Cox | -.2861 | 1.0000 | .1736 | .0888 | .4522 | .4418 | .3465 | .0202 | -.2780 | -.1363 | -.0311 |
|  | N=54 | N=54 | N=54 | N=50 | N=54 | N=54 | N=51 | N=54 | N=21 | N=25 | N=26 |
|  | p=.036 | p= --- | p=.209 | p=.540 | p=.001 | p=.001 | p=.013 | p=.885 | p=.222 | p=.516 | p=.880 |
| 5-fdC Box-Cox | .2368 | .1736 | 1.0000 | .5361 | .6277 | .5801 | .1631 | -.1244 | -.2463 | -.0448 | .0793 |
|  | N=64 | N=54 | N=65 | N=58 | N=64 | N=65 | N=61 | N=65 | N=25 | N=33 | N=35 |
|  | p=.060 | p=.209 | p= --- | p=.000 | p=.000 | p=.000 | p=.209 | p=.324 | p=.235 | p=.804 | p=.651 |
| 5-cadC Box-Cox | -.0618 | .0888 | .5361 | 1.0000 | .3344 | .4541 | .1850 | -.2006 | -.2066 | .0568 | .0721 |
|  | N=58 | N=50 | N=58 | N=58 | N=58 | N=58 | N=54 | N=58 | N=23 | N=31 | N=32 |
|  | p=.645 | p=.540 | p=.000 | p= --- | p=.010 | p=.000 | p=.181 | p=.131 | p=.344 | p=.761 | p=.695 |
| 5-hmdU Box-Cox | -.0001 | .4522 | .6277 | .3344 | 1.0000 | .3718 | .3009 | -.0567 | -.4236 | .0009 | .1250 |
|  | N=64 | N=54 | N=64 | N=58 | N=65 | N=65 | N=61 | N=65 | N=25 | N=33 | N=35 |
|  | p=.999 | p=.001 | p=.000 | p=.010 | p= --- | p=.002 | p=.018 | p=.654 | p=.035 | p=.996 | p=.474 |
| 8-oxodG Box-Cox | -.1665 | .4418 | .5801 | .4541 | .3718 | 1.0000 | .3722 | -.0555 | .2620 | -.0078 | -.0519 |
|  | N=64 | N=54 | N=65 | N=58 | N=65 | N=66 | N=62 | N=66 | N=25 | N=33 | N=35 |
|  | p=.188 | p=.001 | p=.000 | p=.000 | p=.002 | p= --- | p=.003 | p=.658 | p=.206 | p=.966 | p=.767 |
| ascorbate Box-Cox | -.1314 | .3465 | .1631 | .1850 | .3009 | .3722 | 1.0000 | .1284 | -.1879 | -.1728 | -.1280 |
|  | N=60 | N=51 | N=61 | N=54 | N=61 | N=62 | N=62 | N=62 | N=25 | N=31 | N=33 |
|  | p=.317 | p=.013 | p=.209 | p=.181 | p=.018 | p=.003 | p= --- | p=.320 | p=.368 | p=.353 | p=.478 |
| Re tinol | .2069 | .0202 | -.1244 | -.2006 | -.0567 | -.0555 | .1284 | 1.0000 | .1421 | .0133 | -.1832 |
|  | N=64 | N=54 | N=65 | N=58 | N=65 | N=66 | N=62 | N=67 | N=25 | N=33 | N=35 |
|  | p=.101 | p=.885 | p=.324 | p=.131 | p=.654 | p=.658 | p=.320 | p= --- | p=.498 | p=.941 | p=.292 |
| α-tocopherol | .0402 | -.0150 | -.2297 | -.2319 | -.0110 | -.1961 | -.1160 | .4122 | 1.0000 | -.0758 | -.0030 |
|  | N=64 | N=54 | N=65 | N=58 | N=65 | N=66 | N=62 | N=67 | N=67 | N=25 | N=33 |
|  | p=.752 | p=.914 | p=.066 | p=.080 | p=.931 | p=.115 | p=.369 | p=.001 | p= --- | p=.719 | p=.987 |
| TET1 expression Box-Cox | .2373 | -.2780 | -.2463 | -.2066 | -.4236 | .2620 | -.1879 | .1421 | 1.0000 | .2035 | .3246 |
|  | N=25 | N=21 | N=25 | N=23 | N=25 | N=25 | N=25 | N=25 | N=26 | N=24 | N=26 |
|  | p=.253 | p=.222 | p=.235 | p=.344 | p=.035 | p=.206 | p=.368 | p=.498 | p= --- | p=.340 | p=.106 |
| TET2 expression Box-Cox | -.1587 | -.1363 | -.0448 | .0568 | .0009 | -.0078 | -.1728 | .0133 | .2035 | 1.0000 | .6803 |
|  | N=33 | N=25 | N=33 | N=31 | N=33 | N=33 | N=31 | N=33 | N=24 | N=34 | N=34 |
|  | p=.378 | p=.516 | p=.804 | p=.761 | p=.996 | p=.966 | p=.353 | p=.941 | p=.340 | p= --- | p=.000 |
| TET3 expression Box-Cox | .0821 | -.0311 | .0793 | .0721 | .1250 | -.0519 | -.1280 | -.1832 | .3246 | .6803 | 1.0000 |
|  | N=35 | N=26 | N=35 | N=32 | N=35 | N=35 | N=33 | N=35 | N=26 | N=34 | N=36 |
|  | p=.639 | p=.880 | p=.651 | p=.695 | p=.474 | p=.767 | p=.478 | p=.292 | p=.106 | p=.000 | p= --- |

Figure S5 Correlations in polyp group


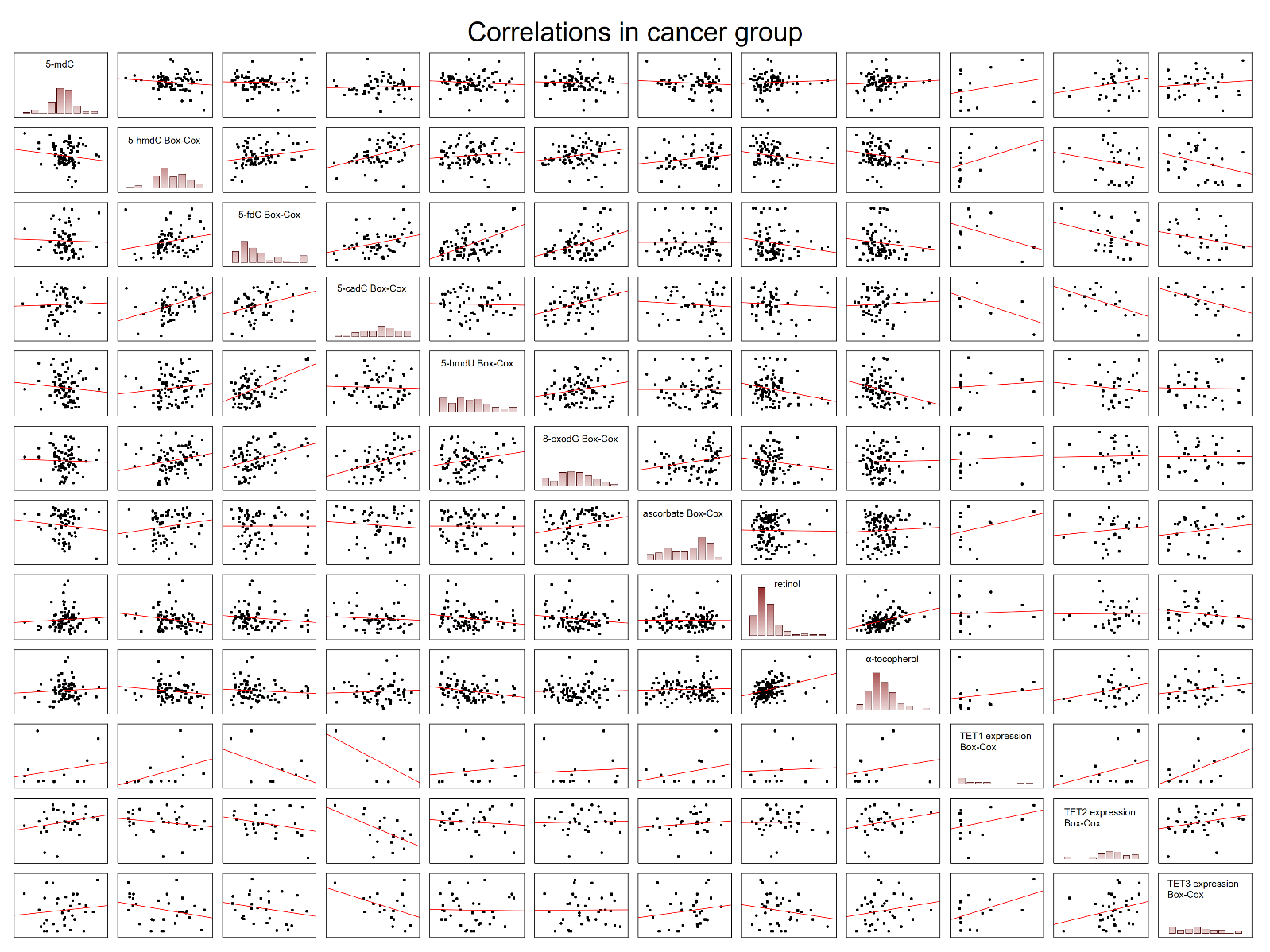


|  | 5-mdC | 5-hmdC Box-Cox | 5-fdC Box-Cox | 5-cadC Box-Cox | 5-hmdU Box-Cox | 8-oxodG Box-Cox | ascorbate Box-Cox | retinol | TET1 expression Box-Cox | TET2 expression Box-Cox | TET3 expression Box-Cox |
| --- | --- | --- | --- | --- | --- | --- | --- | --- | --- | --- | --- |
| 5-mdC | 1.0000 | -.1363 | -.0318 | .0372 | -.0969 | -.0337 | -.1068 | .0653 | .2301 | .2384 | .1182 |
|  | N=85 | N=81 | N=73 | N=55 | N=83 | N=80 | N=68 | N=81 | N=13 | N=32 | N=34 |
|  | p= --- | p=.225 | p=.789 | p=.787 | p=.384 | p=.767 | p=.386 | p=.563 | p=.449 | p=.189 | p=.505 |
| 5-hmdC Box-Cox | -.1363 | 1.0000 | .2159 | .4055 | .1276 | .2260 | .1770 | -.1813 | .4245 | -.1817 | -.2870 |
|  | N=81 | N=84 | N=73 | N=55 | N=83 | N=80 | N=67 | N=80 | N=12 | N=28 | N=30 |
|  | p=.225 | p= --- | p=.067 | p=.002 | p=.250 | p=.044 | p=.152 | p=.107 | p=.169 | p=.355 | p=.124 |
| 5-fdC Box-Cox | -.0318 | .2159 | 1.0000 | .3124 | .5632 | .3954 | .0007 | -.1509 | -.4704 | -.2877 | -.2303 |
|  | N=73 | N=73 | N=85 | N=54 | N=81 | N=78 | N=70 | N=81 | N=10 | N=25 | N=27 |
|  | p=.789 | p=.067 | p= --- | p=.021 | p=.000 | p=.000 | p=.996 | p=.179 | p=.170 | p=.163 | p=.248 |
| 5-cadC Box-Cox | .0372 | .4055 | .3124 | 1.0000 | -.0285 | .3909 | -.0943 | -.0609 | -.6069 | -.5324 | -.4279 |
|  | N=55 | N=55 | N=54 | N=59 | N=59 | N=58 | N=45 | N=57 | N=6 | N=18 | N=19 |
|  | p=.787 | p=.002 | p=.021 | p= --- | p=.830 | p=.002 | p=.538 | p=.653 | p=.201 | p=.023 | p=.068 |
| 5-hmdU Box-Cox | -.0969 | .1276 | .5632 | -.0285 | 1.0000 | .2247 | -.0028 | -.2084 | .1139 | -.1114 | -.0222 |
|  | N=83 | N=83 | N=81 | N=59 | N=93 | N=88 | N=75 | N=89 | N=13 | N=32 | N=34 |
|  | p=.384 | p=.250 | p=.000 | p=.830 | p= --- | p=.035 | p=.981 | p=.050 | p=.711 | p=.544 | p=.901 |
| 8-oxodG Box-Cox | -.0337 | .2260 | .3954 | .3909 | .2247 | 1.0000 | .2425 | -.1514 | .0621 | .0239 | .0051 |
|  | N=80 | N=80 | N=78 | N=58 | N=88 | N=91 | N=74 | N=88 | N=13 | N=32 | N=34 |
|  | p=.767 | p=.044 | p=.000 | p=.002 | p=.035 | p= --- | p=.037 | p=.159 | p=.840 | p=.897 | p=.977 |
| ascorbate Box-Cox | -.1068 | .1770 | .0007 | -.0943 | -.0028 | .2425 | 1.0000 | -.0124 | .2987 | .1196 | .1858 |
|  | N=68 | N=67 | N=70 | N=45 | N=75 | N=74 | N=115 | N=115 | N=12 | N=29 | N=33 |
|  | p=.386 | p=.152 | p=.996 | p=.538 | p=.981 | p=.037 | p= --- | p=.896 | p=.346 | p=.536 | p=.301 |
| retinol | .0653 | -.1813 | -.1509 | -.0609 | -.2084 | -.1514 | -.0124 | 1.0000 | .0520 | .0115 | -.1848 |
|  | N=81 | N=80 | N=81 | N=57 | N=89 | N=88 | N=115 | N=136 | N=14 | N=33 | N=37 |
|  | p=.563 | p=.107 | p=.179 | p=.653 | p=.050 | p=.159 | p=.896 | p= --- | p=.860 | p=.949 | p=.273 |
| α-tocopherol | .0669 | -.1566 | -.1098 | .0544 | -.2481 | .0236 | .0460 | .3399 | 1.0000 | .1896 | .2613 |
|  | N=81 | N=80 | N=81 | N=57 | N=89 | N=88 | N=115 | N=136 | N=136 | N=14 | N=33 |
|  | p=.553 | p=.165 | p=.329 | p=.688 | p=.019 | p=.827 | p=.626 | p=.000 | p= --- | p=.516 | p=.142 |
| TET1 expression Box-Cox | .2301 | .4245 | -.4704 | -.6069 | .1139 | .0621 | .2987 | .0520 | 1.0000 | .3419 | .5073 |
|  | N=13 | N=12 | N=10 | N=6 | N=13 | N=13 | N=12 | N=14 | N=15 | N=13 | N=15 |
|  | p=.449 | p=.169 | p=.170 | p=.201 | p=.711 | p=.840 | p=.346 | p=.860 | p= --- | p=.253 | p=.054 |
| TET2 expression Box-Cox | .2384 | -.1817 | -.2877 | -.5324 | -.1114 | .0239 | .1196 | .0115 | .3419 | 1.0000 | .2796 |
|  | N=32 | N=28 | N=25 | N=18 | N=32 | N=32 | N=29 | N=33 | N=13 | N=35 | N=35 |
|  | p=.189 | p=.355 | p=.163 | p=.023 | p=.544 | p=.897 | p=.536 | p=.949 | p=.253 | p= --- | p=.104 |
| TET3 expression Box-Cox | .1182 | -.2870 | -.2303 | -.4279 | -.0222 | .0051 | .1858 | -.1848 | .5073 | .2796 | 1.0000 |
|  | N=34 | N=30 | N=27 | N=19 | N=34 | N=34 | N=33 | N=37 | N=15 | N=35 | N=39 |
|  | p=.505 | p=.124 | p=.248 | p=.068 | p=.901 | p=.977 | p=.301 | p=.273 | p=.054 | p=.104 | p= --- |

Figure S6 Correlations in cancer group

Figure S7 Multiple correlations between TET3 mRNA expression, plasma ascorbate concentration and levels of endogenous DNA modifications


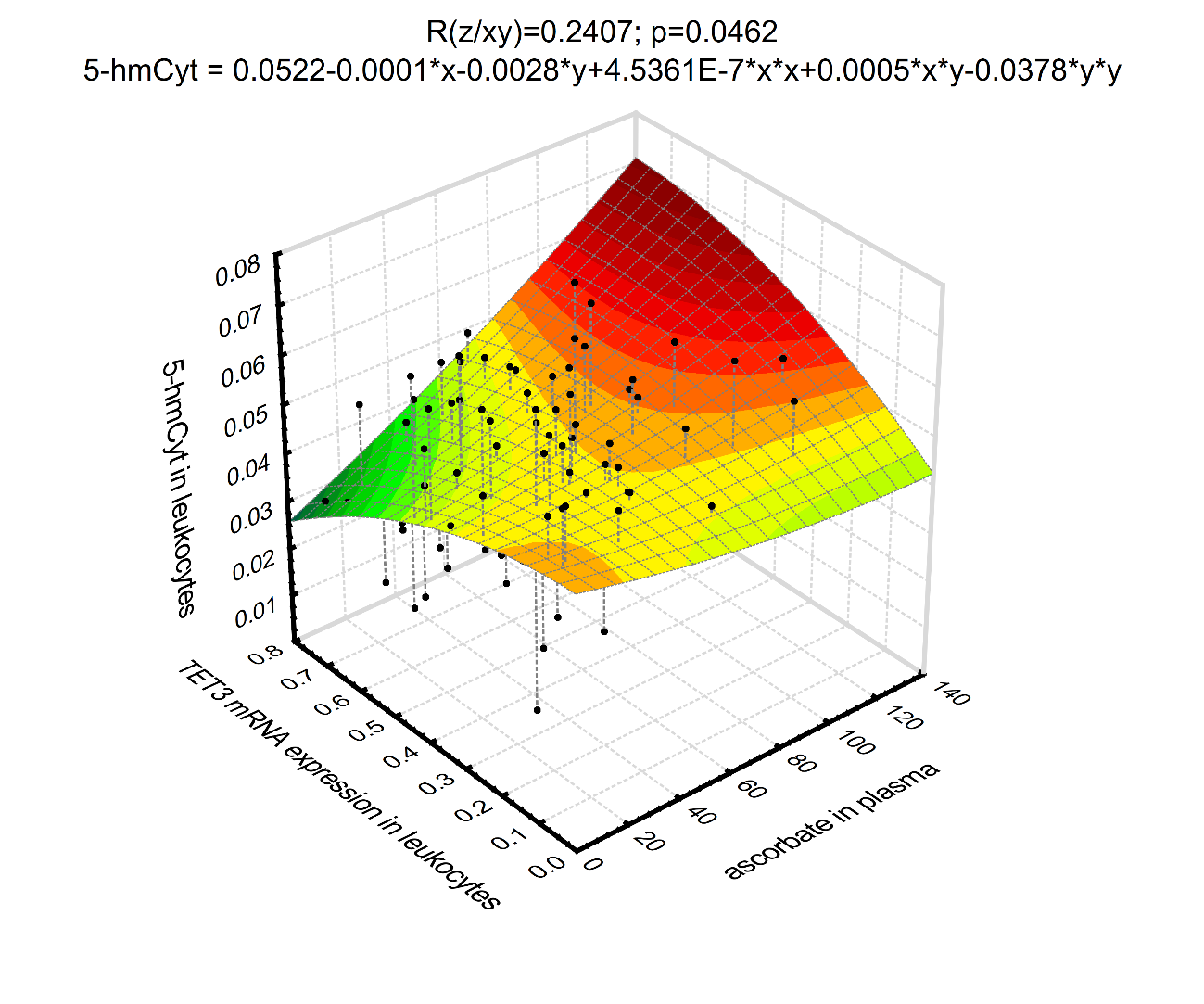

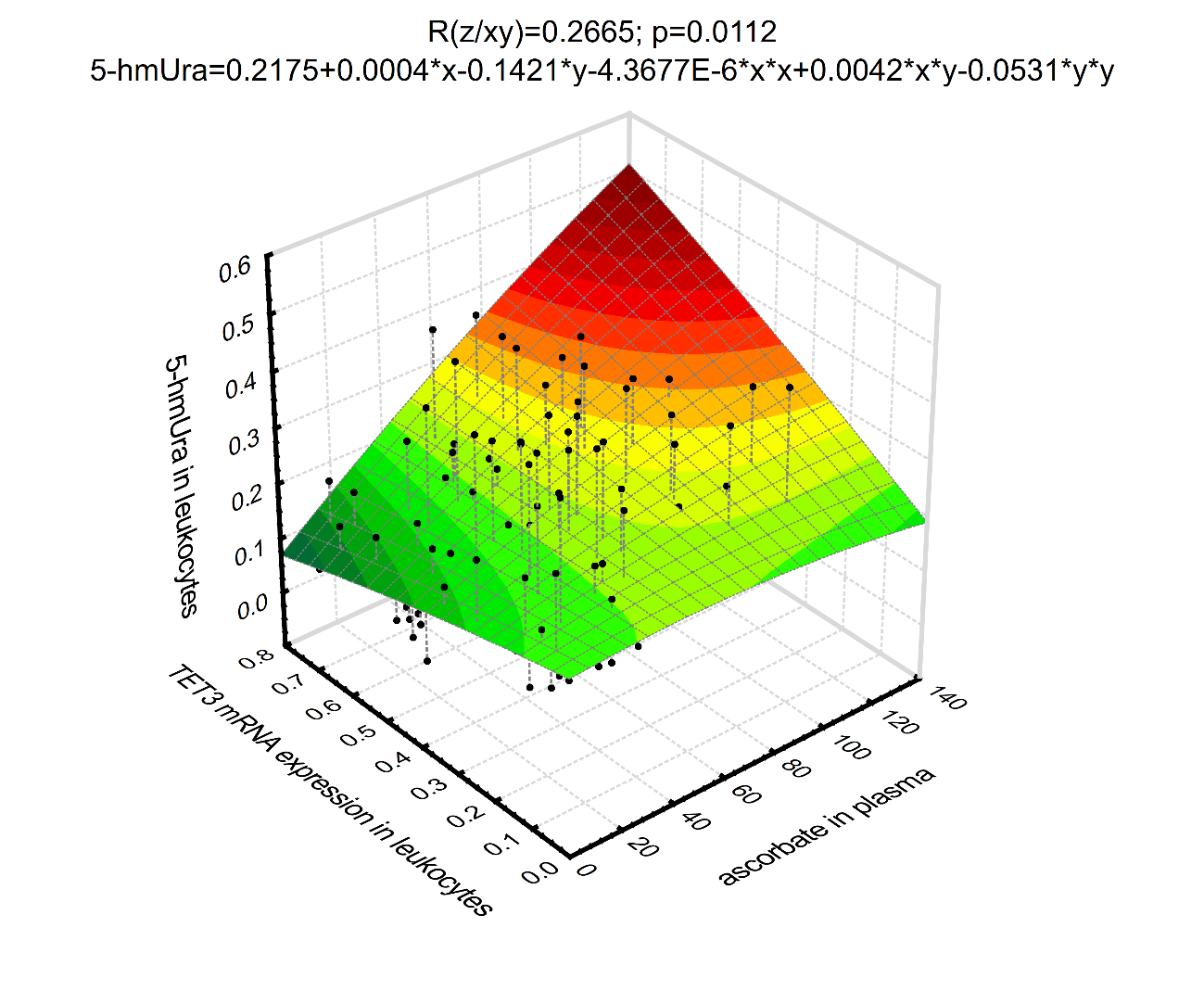


Figure S8 Multiple correlations between TET2 mRNA expression, plasma ascorbate concentration and levels of endogenous DNA modifications


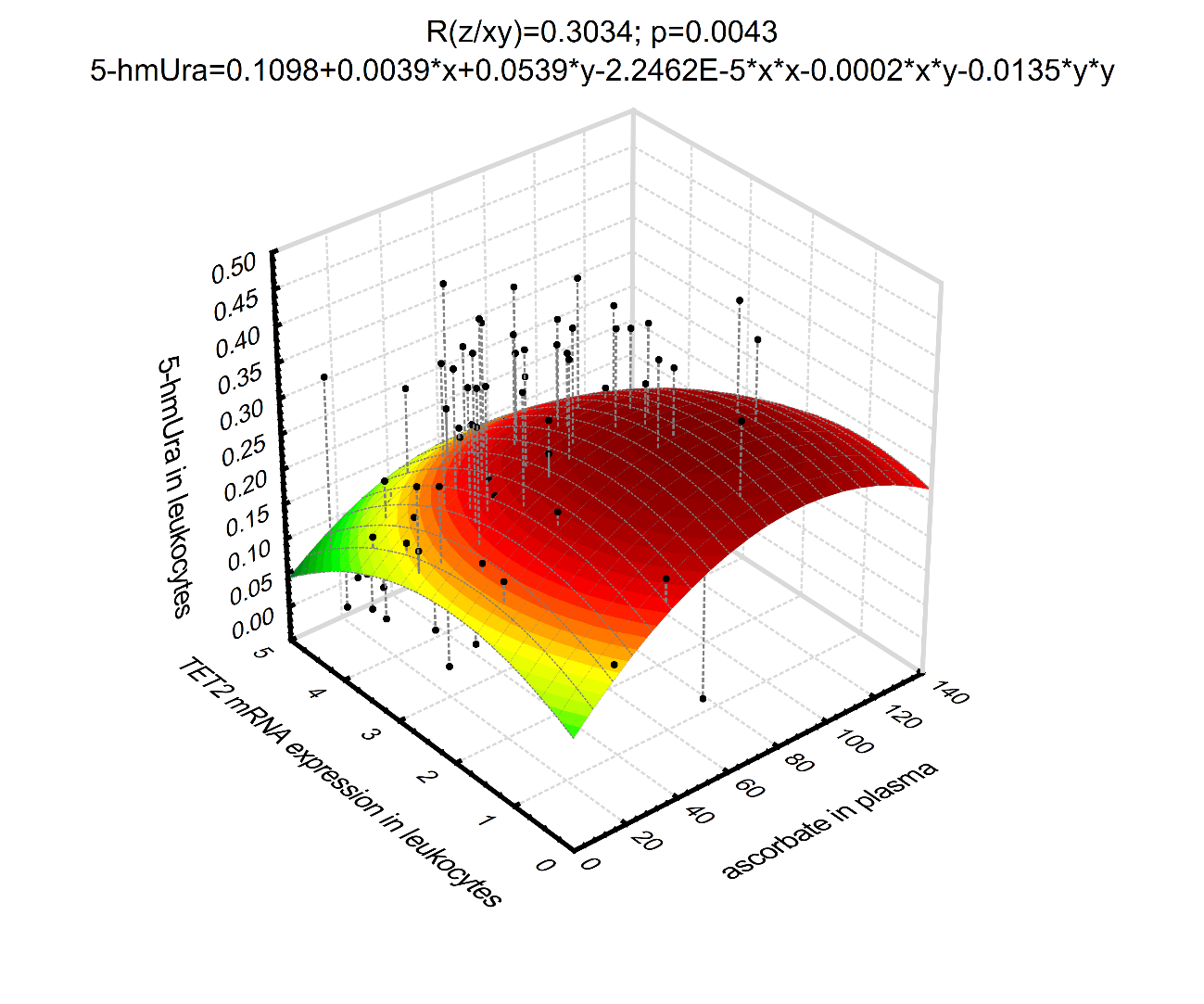

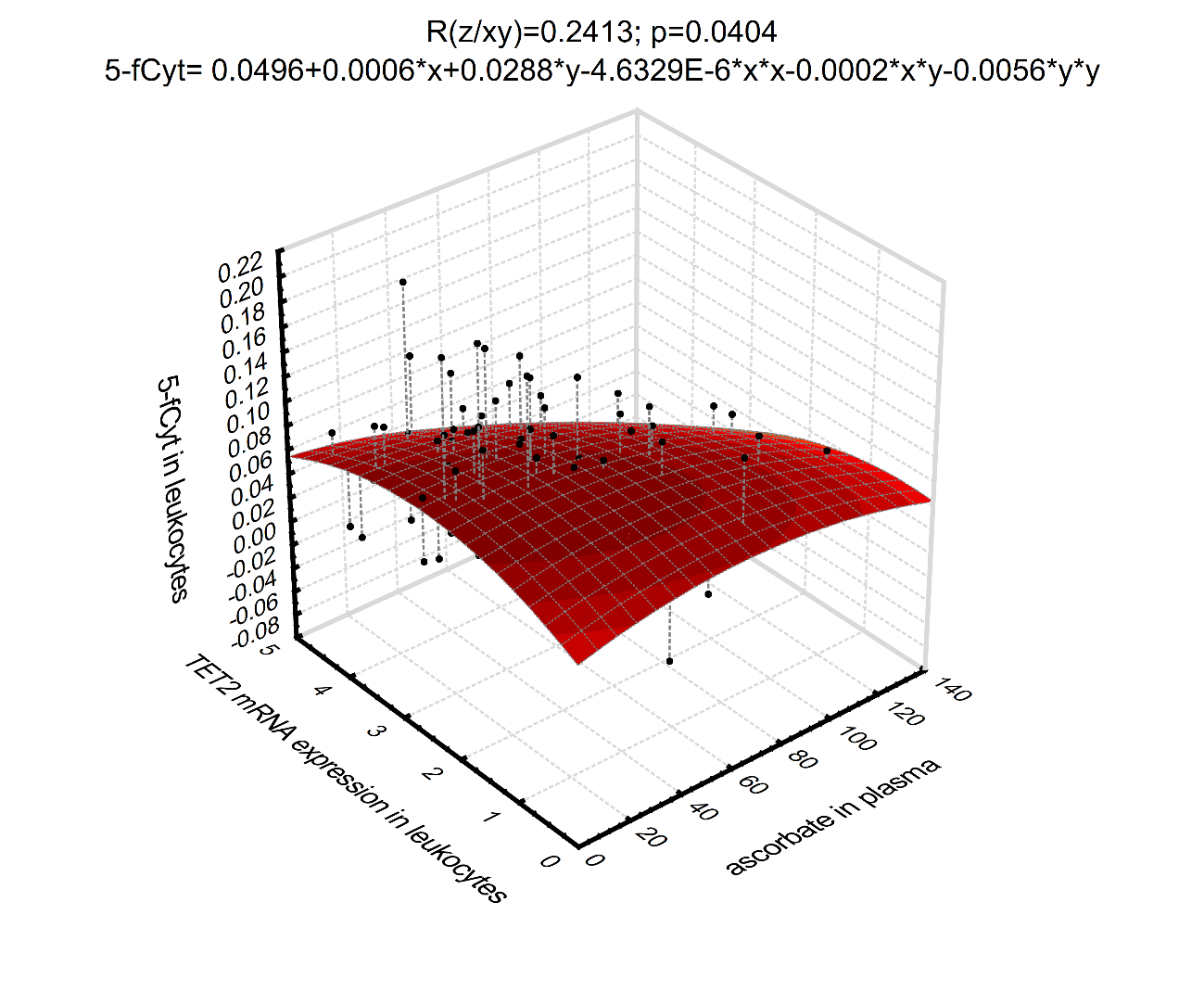


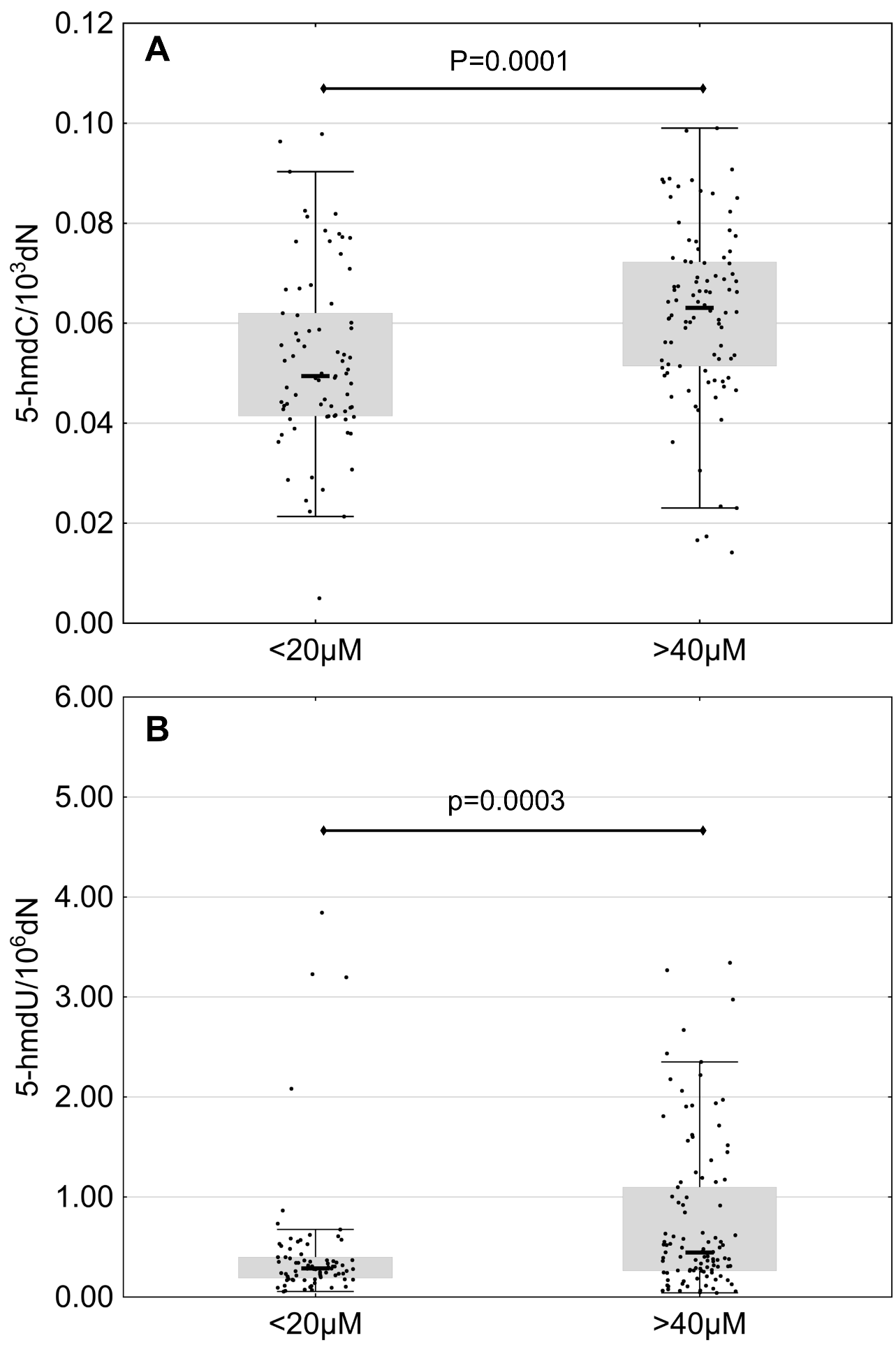
Figure S9 Selected, statistically significant associations between the levels of DNA modifications and plasma concentrations of ascorbate in individuals with concentration above 40 µM and in the group with concentration below 20µM.

***Additional method – gene expression analysis***

Concentration and purity of RNA aliquots were verified spectrophotometrically with NanoDrop 2000 (Thermo Scientific). A_260_/A_280_ ratio was used as an indicator of protein contamination, and A_260_/A_230_ ratio as a measure of contamination with polysaccharides, phenol and/or chaotropic salts. Quality and integrity of total RNA were assessed by visualization of 28S/18S/5.8S rRNA band pattern in a 1.2% agarose gel. Non-denaturing electrophoresis was carried out at 95 V for 20 min in TBE buffer (Tris – Boric Acid – EDTA). The gel was stained with ethidium bromide or SimplySafe and visualized using GBox EF Gel Documentation System (SynGene).

The samples with RNA concentrations greater than 50 ng/μl were qualified for further analysis. 0.5 microgram of total RNA from each sample (in 20-μl volume) was used for cDNA synthesis by reverse transcription with High-Capacity cDNA Reverse Transcription Kit (Applied Biosystems, catalog no. 43-688-14), according to the manufacturer’s instruction. The reaction was carried out with Mastercycler Nexus Gradient thermocycler (Eppendorf). To exclude contamination with genomic DNA, reverse transcriptase reaction included also a negative control. cDNA was either used for qPCR setup immediately after obtaining, or stored at −20°C.

Three gene transcripts, *TET1, TET2* and *TET3*, were analyzed by relative quantitative RT-PCR (RT-qPCR) with relevant primers and short hydrolysis probes substituted with Locked Nucleic Acids from the Universal Probe Library (UPL, Roche). The probes were labeled with fluorescein (FAM) at the 5'-end and with a dark quencher dye at the 3'-end. Real-time PCR mixes (in 20-μl volumes) were prepared from cDNA following the standard procedures for LightCycler480 Probes Master (Roche), provided with the reagent set. The reactions were carried out on 96-well plates. Aside from the proper samples, each plate included also no-template control and no-RT control. Quantitative real-time PCR was carried out with LightCycler 480 II, using the following cycling parameters: 10 s at 95°C, followed by 45 repeats, 10 s each, at 95°C, 30 s at 58°C, and finally, 1 s at 72°C with acquisition mode (parameters of wavelength excitation and detection equal 465 nm and 510 nm, respectively). The reaction for each gene was standardized against a standard curve, to estimate amplification efficiency. Standardization procedure included preparation of 10-fold serial dilutions with controlled relative amount of targeted template. The efficiency of amplification was assessed based on a slope of the standard curve. Standard dilutions were amplified in separate wells, but within the same run. In order to confirm the obtained results of TETs mRNA expression analysis, the experiment was repeated using other probes and primers (Table S3) on 45 randomly chosen samples. Very high correlations and similar Ct were found (Figure S10).

| Gene | Forward primer sequence | Reverse primer sequence | UPL | Probe sequence |
| --- | --- | --- | --- | --- |
| *TET1* | 5’-Cagccatcagatctgtaagaaaag-3’ | 5’-ggcctcttgttttcctttataacc-3’ | #17 | aggagctg |
| *TET2* | 5’-caggctaaacagttggcaga-3’ | 5’-ctgctggggtctctgctg-3’ | #83 | gcagccac |
| *TET3* | 5’-aaggagcggctgtcaaga-3’ | 5’-tacactggccctgagtccaT-3’ | #85 | tccaggtc |

Table. S3 Alternative primers and short hydrolysis probes used for validation of previous TETs mRNA expression analysis


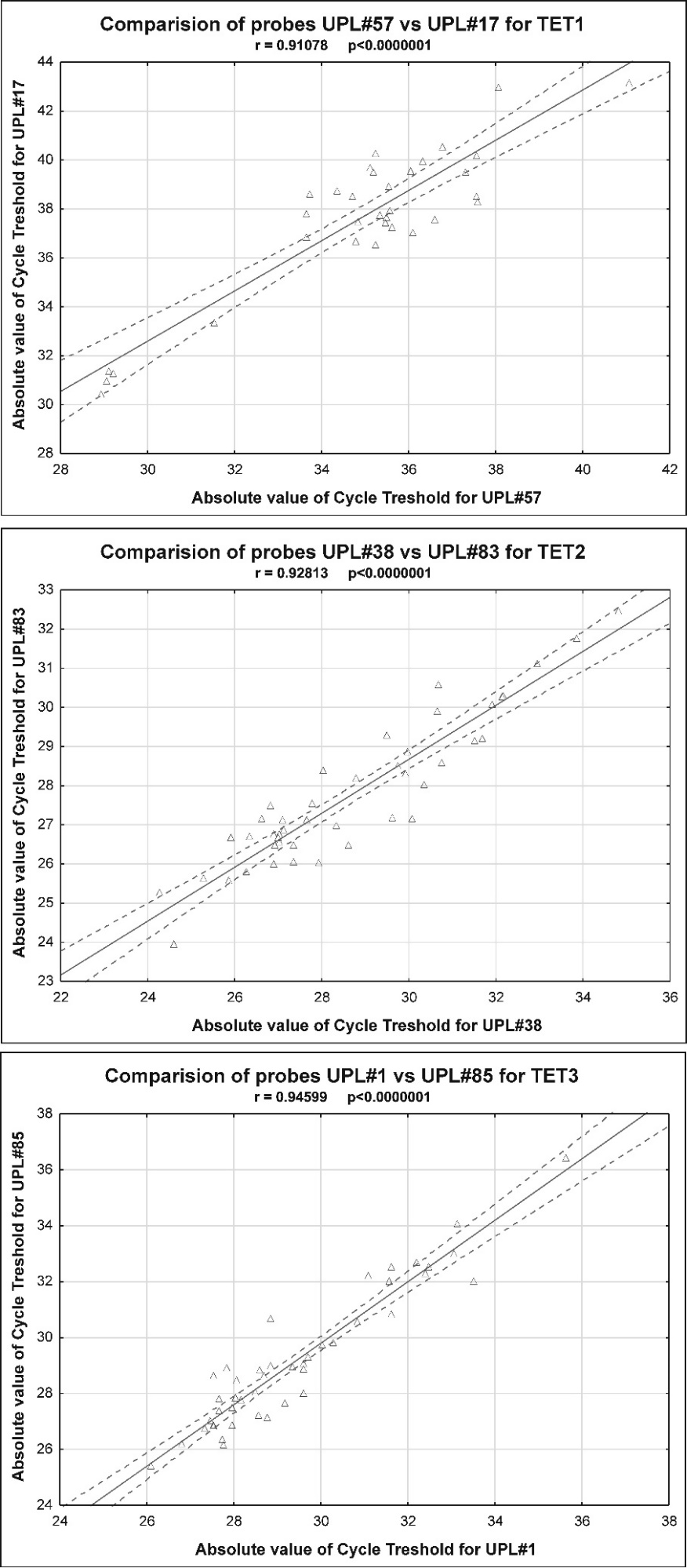


Figure S10 Correlations between different primers and short hydrolysis probes used for TETs mRNA expression analysis
